# A Predictive Model for Benchmarking the Performance of Algorithms for Fake and Counterfeit News Classification in Global Networks

**DOI:** 10.3390/s24175817

**Published:** 2024-09-07

**Authors:** Nureni Ayofe Azeez, Sanjay Misra, Davidson Onyinye Ogaraku, Ademola Philip Abidoye

**Affiliations:** 1 Department of Computer Sciences, University of Lagos, Lagos 100213, Nigeria; nazeez@unilag.edu.ng (N.A.A.); davidson.ogaraku@gmail.com (D.O.O.); 2 Institute for Energy Technology, 1777 Halden, Norway; 3 Department of Computer Science and Information Technology, Clayton State University, Morrow, GA 30260, USA; aabidoye@atlantatech.edu

**Keywords:** fake news, sensors, classification, ensemble, metrics, algorithm, learning, evaluation

## Abstract

The pervasive spread of fake news in online social media has emerged as a critical threat to societal integrity and democratic processes. To address this pressing issue, this research harnesses the power of supervised AI algorithms aimed at classifying fake news with selected algorithms. Algorithms such as Passive Aggressive Classifier, perceptron, and decision stump undergo meticulous refinement for text classification tasks, leveraging 29 models trained on diverse social media datasets. Sensors can be utilized for data collection. Data preprocessing involves rigorous cleansing and feature vector generation using TF-IDF and Count Vectorizers. The models’ efficacy in classifying genuine news from falsified or exaggerated content is evaluated using metrics like accuracy, precision, recall, and more. In order to obtain the best-performing algorithm from each of the datasets, a predictive model was developed, through which SG with 0.681190 performs best in Dataset 1, BernoulliRBM has 0.933789 in Dataset 2, LinearSVC has 0.689180 in Dataset 3, and BernoulliRBM has 0.026346 in Dataset 4. This research illuminates strategies for classifying fake news, offering potential solutions to ensure information integrity and democratic discourse, thus carrying profound implications for academia and real-world applications. This work also suggests the strength of sensors for data collection in IoT environments, big data analytics for smart cities, and sensor applications which contribute to maintaining the integrity of information within urban environments.

## 1. Introduction

Online social media platforms have become increasingly influential in a time when digital communication and information exchange are advancing quickly. These platforms have emerged as the main avenues for the broadcast of news, presenting both previously unheard-of potential and difficulties. The frequency and effects of fake news, which is purposefully false or misleading material presented as news, are among the most urgent problems [1].

The emergence of false news has wide-ranging effects. The public’s confidence in media organizations and democratic institutions is also weakened, in addition to the credibility of information sources. Misinformation can spread like wildfire over social media networks, confusing the audience and possibly influencing their opinions and decisions [2]. Misinformation is frequently fueled by clickbait, political goals, or sensationalism. Researchers and professionals have turned to cutting-edge technology, such as supervised artificial intelligence algorithms, to assist in identifying false information within the dynamic and linked landscape of online social media in order to address this issue.

This study proposes a two-phased detection methodology designed to identify instances of fake news within the realm of social media. The recommended framework integrates supervised artificial intelligence algorithms with text analysis techniques, forming an innovative approach. In the initial stage of the project, text mining methodologies are employed to analyze a dataset comprising internet news. The primary objective of these text analysis techniques is to extract structured information from unstructured news stories.

In the subsequent phase, a total of twenty-nine algorithms for supervised artificial intelligence are enlisted to perform the task of classifying fake news from legitimate news. These algorithms encompass a diverse range, including “lbfgs” logistic regression, “liblinear” logistic regression, “newton-cg” logistic regression, “sag” logistic regression, Random Forest, perceptron, Ridge Classifier, CatBoost, Nearest Centroid, Stochastic Gradient Descent (SGD), SVC (kernel = “linear”, C = 0.025), SVC (gamma = 2, C = 1), LinearSVC, ZeroR, decision tree, Passive Aggressive, Extra Tree, Random Patches, voting, stacked generalization, multilayer perceptron (MLP), Bernoulli RBM, AdaBoost, gradient boosting, ordinal learning model, XGBoost, decision stump, Complement Naïve Bayes, Multinomial Naïve Bayes.

To ensure robustness, these supervised algorithms are subjected to rigorous training and testing using four distinct datasets. Through this comprehensive approach, this study aims to effectively distinguish between bogus news and authentic news within the dynamic and complex landscape of social media.

This research has achieved uniqueness by creating a predictive model that can easily choose the best algorithms and maximize their performance. Furthermore, the model effectively improves evaluation by offering a consistent method for assessing algorithmic performance, which in turn identifies regions in which algorithms perform well or poorly. It assists in identifying algorithms that stop the spread of bogus news before it causes havoc by detecting it early. By detecting fake news before it goes viral, real-time prediction research work not only enhances model accuracy by collecting the newest trends and patterns but also allows for quick action against fake news, decreasing its spread. Rapid incident reaction and mitigation are further facilitated by the real-time forecast. In order to classify fake and counterfeit news, this work allows developers and researchers to construct more dependable and effective solutions by benchmarking numerous algorithms. This study clarifies how decisions are made and explains why certain news stories are classified as either positive or negative. Furthermore, it is quite sure that the models can support a variety of datasets, techniques, and use cases. Among the innovations of this research endeavor are these succinct explanations and others.

The following are the major contributions of our research:

- Development of predictive models for benchmarking the performance of the algorithms used for fake and counterfeit news classification in global networks.
- Benchmarking of multiple algorithms: In order to determine which techniques are the most efficient, this work has been able to benchmark the performance of multiple algorithms.
- Real-time prediction: The method can identify bogus and fraudulent news in real time, enabling prompt alerting, awareness raising, and intervention.
- Interpretability and explainability: This study sheds light on the decision-making process and explains why some news items are categorized as either positive or negative (fake or counterfeit).
- Constant learning and adaptation: By incorporating mechanisms for ongoing learning and adaptation, this work can remain effective even in the face of changing techniques used by fake and counterfeit news sources.
- Provision of a platform for internet users to identify fake news and subsequently prevent them from being victims of fake and fraudulent news.
- A searchlight for internet users to be selective on where to source reliable real-time information.

The following are some real-world uses for algorithms designed to classify false and counterfeit news on international networks:

1. Social media companies: By incorporating algorithms to identify and eliminate false material, they can stop the spread of false information.
2. News aggregators and fact-checking websites: These websites automate the process of fact-checking news stories by using algorithms.
3. Search engines: Users are guaranteed to receive reliable information by applying algorithms that demote or delete phony news from search results.
4. Online advertising platforms employ algorithms to thwart the display of advertisements on fake news websites, diminishing the sources of income for disinformation.
5. Using algorithms to track and examine the dissemination of false information, government and law enforcement organizations can make informed decisions about enforcement and policy.
6. Media outlets and journalists: Upholding journalistic integrity by using algorithms to validate sources and identify false information.

### Structure of the Paper

The entire paper is as follows: Section 1 presents the Introduction, while the summary of related works is presented in Section 2. The materials and methods used are detailed in Section 3. The results obtained in the course of experimentation are provided in Section 4, while the predictions of the best-performing algorithms are comprehensively presented in Section 5. The discussion around the results as well as the concluding parts is provided in Section 6, respectively. 

## 2. Related Works

In 2022, Shaina and Chen suggested a novel architecture for detecting fake news that was intended to overcome issues like the early detection of bogus news and the lack of labeled data needed to train the detection model [3]. This system uses data from the report articles and the social setting to identify fake news. It is based on a transformer design and was influenced by the BART architecture. The model’s encoder blocks carry out the representation learning task. The issues of early fake news detection are further helped by the decoder blocks, which forecast future behavior based on historical observations. Compared to autoencoding models (exBake and BERT), autoregressive models (Grover and GPT-2) performed better for early detection. ExBAKE, FANG, 2-Stage Transf., Declare, TriFN, and VGCN-BERT all showed improved performance in later time steps, according to the authors. This behavior was attributed to longer learning time. The performance of the LG, TextCNN, and XGBoost was noted to have been inferior to that of the other baselines.

An effective deep diffusive neural network model for fake news identification named FakeDetector is proposed in the publication “FakeDetector: Effective Fake News Detection with Deep Diffusive Neural Network” [4]. By extracting explicit and latent elements from textual data, the model simultaneously learns subjects, writers, and news article representations. This strategy is based on the finding that looking into correlations between news pieces, their subjects or topics, and their authors or distributors can help with false news identification [5].

In 2019, Benamira et al. [6] examined the use of semi-supervised learning and graph neural networks for the identification of false news. The requirement for efficient detection techniques that can use both labeled and unlabeled data to increase accuracy is what spurred this strategy. In order to handle data represented in graph domains, neural network models known as “graph neural networks” (GNNs) were developed [7]. They have been used for many different purposes, such as graph node categorization. Both labeled and unlabeled data are used in semi-supervised learning, a learning methodology, to train models. Graph neural networks and semi-supervised learning have the potential to enhance fake news identification. GNNs may capture interactions and dependencies between nodes by utilizing the graph structure of social networks or other data representations, which might be helpful for spotting patterns of fake news propagation. Additionally, semi-supervised learning with unlabeled data enables the model to learn from a bigger dataset, potentially improving its capacity to reliably identify bogus news. The experimental results, in the authors’ opinion, showed that the suggested methodology performed better than conventional classification algorithms, particularly when trained on a small sample of tagged articles.

A benchmark framework for examining and discussing machine/deep learning methods used in fake news detection was given by Galli et al. [8] in 2022. Their framework intends to overcome the difficulties associated with fake news identification, such as the variety of subjects and language elaborations employed in its creation. It offered a consistent evaluation framework for contrasting the effectiveness of various detection models. The authors investigated how different elements, including textual, social, and network-based features, may be used to identify bogus news. They evaluated the effectiveness of several methods and offered details on the advantages and disadvantages of every strategy. Three datasets—FakeNews, a big, unbalanced dataset; and PHEME and LIAR, two smaller datasets—were used for these analyses. First, different machine learning models (logistic regression, decision tree, SVC, and Random Forest) were compared; the results showed that logistic regression was the most successful model in terms of efficacy and efficiency measures. In contrast to other models, logistic regression has several advantages, such as simplicity of interpretation, speed of implementation, and few tuning parameters. B.E.R.T achieved the greatest overall results because it conducted context-based word-level embedding while being challenging to train. By combining multimedia and content analysis, a multimodal technique has been further developed to perform a false image classification. This approach yielded the greatest results in terms of recall, accuracy, F1, and precision using multimedia data.

Using the swarming traits of fake news, the FakeSwarm fake news recognition system was introduced in 2023 [9]. The authors used three distinct swarm feature types—metric representation, principal component analysis, and location encoding—to demonstrate the importance of considering swarming characteristics in the identification of false news. The ISOT fake news dataset was used by the authors to carry out their analysis. There are 23,481 phony news pieces and 21,417 true news articles in the dataset, which contains news stories from 2015 to 2018. The fake news pieces were gathered from several sites that fact-checking agencies like Politifact and Wikipedia had identified as false. On the other hand, reliable content came through crawling from Reuters.com. The authors’ examination of the available data revealed that combining all three swarm feature categories produced an excellent accuracy of more than 97% and an F1 score.

A two-step method was proposed by [10] for identifying fake news on social media. Preprocessing the data was the initial step in the method’s procedure to convert unstructured datasets into structured datasets. The news texts and other texts in the dataset are vectorized using the Document-Term Matrix and the acquired TF weighting algorithm. In the second stage, 23 supervised AI algorithms were applied to the dataset, which had previously been text-mined and organized into a structured format. This study used publicly available datasets to empirically test twenty-three intelligent classification techniques. The four evaluation metrics (i.e., recall, accuracy, F-measure, and precision) were then used to compare these classification models. The authors claimed that the decision tree method has produced the best mean values in terms of accuracy, precision, and F-measure. In terms of recall metrics, the 1000 value algorithms ZeroR, CVPS, and WIHW appeared to be the best.

Wang et al. [11] introduced a novel fine-grained multimodal fusion network (FMFN) to fully fuse textual and visual information for the purpose of identifying fake news. When a tweet has both text and an image, word embeddings from the text are extracted using a pretrained language model, and each word embedding can be considered a textual feature. Deep CNNs are utilized to extract various visual aspects from the image. Scaled dot-product attention was used to combine word embeddings from the text with multiple feature vectors representing different aspects of the image. This approach captures the dependencies between textual and visual features more accurately and accounts for correlations between different visual features. The FMFN, which fuses multiple visual features and multiple textual features, learns a joint representation that is superior to that learned by other methods for fusing visual representation and text representation obtained by combining a text representation with a visual representation when identifying fake news, according to the findings of an extensive experiment the authors carried out on a publicly available Weibo dataset.

The Mc-DNN multi-channel deep learning model was introduced by Tembhurne et al. [12] in 2022. It employs and processes news headlines and articles from several channels to distinguish between real and fraudulent news. The performance of Mc-DNN was investigated using the combinations of RNN and CNN, GRU and CNN, LSTM and CNN, BiGRU and CNN, and BiLSTM and CNN. BiLSTM and CNN Mc-DNN were reported to achieve the maximum accuracy of 99.23% and 94.68%, respectively, in the task of false news identification on the ISOT fake news dataset and FND [13]. 

The authors of a previous publication presented an entity debiasing framework (ENDEF), which generalizes fake news detection algorithms to future data by reducing entity bias from a cause-and-effect approach [14]. Based on the causal connection connecting news entities, news contents, and news truthfulness, the authors separately modeled the contribution of each cause (entities and contents) during training. They reduced the direct influence of the entities during the inference stage in order to reduce entity bias. Extensive offline studies on the English and Chinese datasets demonstrate that the proposed method may greatly improve the performance of base false news detectors, and online tests validate its superiority in practice.

According to Murayama et al. [15], the bulk of false news datasets are dependent on a specific time period. As a result, detection models developed using such a dataset struggle to identify unexpected fake news brought about by political and societal changes; they may also produce biased output from the input, such as names of particular people and organizations. Because it is a result of the origination date of news in each dataset, the authors called this diachronic bias. The authors developed masking techniques based on Wiki data to reduce the impact of human names and test whether they strengthen fake news detection algorithms through trials using in-domain and out-of-domain data. Based on their tests, the authors were able to confirm that these masking techniques increased model robustness and accuracy in out-of-domain datasets.

In a different approach, Min et al. [16] formulated the problem of social context-based fake news identification as a diverse graph classification problem and introduced the Post-User Interaction Network (PSIN). This model effectively models the post–post, user–user, and post–user connections in social contexts while maintaining their inherent features through the use of a divide-and-conquer strategy. The authors employed an adversarial topic discriminator for topic-agnostic feature learning in order to broaden the method’s applicability to recently emerging subjects. Extensive experiments demonstrate that the proposed method outperforms SOTA baselines in both on-topic and off-topic scenarios.

Sahoo and Gupta [17] proposed an autonomous detection of false news solution for the Chrome environment to detect fake news on Facebook. The authors used deep learning to analyze the behavior of the account by utilizing a range of Facebook account-related data in addition to other features connected to news articles. These authors claimed that multiple experimental analyses of actual material showed that their intended strategy for detecting false news had more accuracy than the current state-of-the-art methods.

A thorough analysis of the most advanced techniques for identifying malicious users and bots based on the various features suggested in the authors’ unique taxonomy was published in a paper by Shahid et al. [18] in 2022. In order to aid researchers who are new to this subject, the authors discussed numerous important problems and prospective future research areas in an effort to avoid the critical issue of false news detection.

Shu et al. [19] looked at the problem of understanding and using social media user profiles for the identification of fake news in another paper. By tracking users’ sharing habits, the authors were able to identify group representative users who are more likely to spread both false and accurate news. Subsequently, they carried out a comparative study of explicit and implicit profile attributes among various user groups to ascertain their capacity to facilitate the differentiation of false from authentic news. The authors demonstrated the usefulness of user profile features for exploitation with a bogus news categorization problem. The authors further confirmed the effectiveness of these characteristics using feature significance analysis.

In a study by Kaliyar et al. [20], the authors analyzed the substance of the news piece and the echo chambers on social media—groups of people who have the same beliefs—to determine which news was fake. A tensor representing social context—that is, the association between user profiles on social media and news articles—was produced by combining news, user, and community data. The tensor and the news material were integrated to create a representation that included the social context as well as the news information. The suggested method has been tested on the real-world dataset BuzzFeed. The decomposition-derived parameters were used as features in the news classification process. An ensemble machine learning classifier (XGBoost) and a deep neural network model (DeepFakE) were employed for the classification task. According to the scientists, the proposed model (DeepFakE) beats the current techniques for identifying fake news since it uses deep learning on a combination of social context-based qualities and news content as an echo chamber. 

Gedara et al. (2024) adopted the fuzzy transform to achieve the detection of fake news through data reduction. In an attempt to reduce the training period, the architecture of Long Short-Term Memory was adopted. The technique, though with several challenges—the application of a limited number of algorithms—shows a reasonable degree of efficiency when evaluated with the common evaluation metrics [21].

The application of the Similarity-Aware Multimodal Prompt Learning (SAMPLE) technique was adopted by Jiang et al. (2023). This technique, along with the three prompt templates, was used to detect fake news. With the introduction of a similarity-aware fusing method, the accuracy level seems encouraging. The experimentation that was conducted on a few datasets makes the result incomprehensible and unreliable [22].

In an effort to shift focus from the popular approach of textual fake news detection using machine learning approaches, Meel and Vishwakarma in 2021 proposed a multimodal fake news detection technique. The method focuses on four different techniques of multimodal data analysis. The results and comparative evaluation with other similar work show a high degree of accuracy, to the tune of 95.90%. The technique, however, is limited in application as it is now a generalized technique [23,24].

Deepfake is a common technique being used on social media to manipulate the faces of internet users to maliciously commit a crime on behalf of the original owner of the face. Efforts to solve this social media challenge were the focus of [25]. To solve this problem, the authors used a stacking-based ensemble approach, which features a combination of Xception and EfficientNet-B7. A reasonable degree of accuracy was observed. This is an appreciable improvement over the existing solutions, even though there are a limited number of algorithms adopted for ensemble techniques.

## 3. Materials and Methods

This section delves into the heart of the study, focusing on the implementation and methodology of utilizing artificial intelligence (AI) to combat the pervasive issue of fake and counterfeit news within the realm of social media. This section navigates through the intricacies of data collection, preprocessing, and the selection of supervised AI algorithms tailored for text classification tasks. By elucidating the process of model training, validation, and performance evaluation using standard metrics, this chapter offers a comprehensive insight into the strategies employed to discern between authentic and deceptive information circulating across various social media platforms.

### 3.1. Data Collection 

#### 3.1.1. Dataset 1

This dataset of political news was taken out of Kaggle; it was a dataset compiled and provided for a community prediction competition [26]. It consists of 25,116 rows of training data. Each contains the ID, title, author, text, and label columns. And for 5864 rows of test data, each also contains the ID, title, author, and text columns. This dataset was one of four datasets used in training our different machine learning models.

#### 3.1.2. Dataset 2

The second dataset came from the University of Victoria’s Online Academic Community [27]. Thousands of false news and real articles are combined to create this fake news dataset. The dataset was assembled from a number of stories published on both reputable and shady news websites.

#### 3.1.3. Dataset 3

A publicly accessible dataset for identifying false news is called LIAR [28]. POLITIFACT.COM offers a thorough analytical report and links to the original documents for each case, where 12.8K manually labeled brief utterances were gathered over a ten-year period in a variety of circumstances. Research that involves fact-checking can also make use of this dataset. It is interesting to note that this news dataset is orders of magnitude larger than prior, comparable public fake news databases. The LIAR dataset contains 12.8K human-labeled brief statements from POLITIFACT.COM’s API; a POLITIFACT.COM editor verifies each statement.

#### 3.1.4. Dataset 4

The fourth dataset was obtained via the https://zenodo.org/record/4561253 (accessed on 23 January 2024) page on the Zenodo website. For the purpose of detecting fake news in text data, ref. [29] created the WELFake dataset, which has 72,134 news stories with 35,028 true and 37,106 fraudulent news. These are arranged in columns for text, label, serial number, and title. The news heading is represented by the title, the news content is represented by the text, and the label indicates if the news is true or fake (0 being fake and 1 being real). The serial number begins at 0. Just 72,134 of the roughly 78,098 records are accessible according to the dataset. The summary of the datasets is provided in Table 1.

### 3.2. Text Categorization

**Figure 1:**
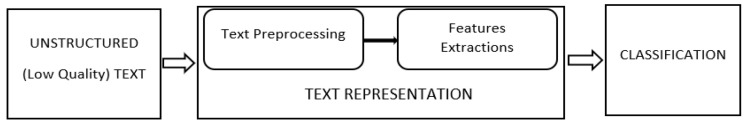
Text classification pipeline [31].

Text categorization also known as text classification is a task in natural language processing (NLP) and machine learning that entails classifying text documents into specified groups or labels according to their content [30]. Text classification aims to automatically classify text documents into one or more specified categories so that organizing, searching, and deriving insights from massive amounts of text data is made simpler. The text classification pipeline is provided in Figure 1 [31].

#### 3.2.1. Text Preprocessing

A series of procedures known as text preprocessing are used to clean and prepare text data for analysis [32]. The following actions are usually involved:

Lowercasing: To guarantee uniformity in text data, convert every text to lowercase. This lessens the likelihood of case sensitivity problems, which facilitates word matching and processing.

Tokenization: Divide the text into discrete words, or tokens, using tokenization. Tokenization plays a crucial role in segmenting sentences or paragraphs into manageable chunks for analysis.

- Removal of Stop Words: Terminating words such as “the”, “and”, “in”, and “of” should be eliminated from the text. For many NLP tasks, these terms are typically not informative.
- Removal of Punctuation: Symbols, special characters, and punctuation are frequently unnecessary for analysis.
- Lemmatization: Reducing words to their most basic or root form is known as lemmatization. Consolidating related words is aided by stemming and lemmatization (e.g., “running” and “ran” both become “run”).
- HTML Tag Removal: You might need to remove HTML tags if the text in your data originates from web pages.
- Text Normalization: Handling email addresses, URLs, and other text-specific patterns may require additional normalization procedures.

#### 3.2.2. Feature Extraction

Textual data must be transformed into vector or numerical representations before being fed into machine learning algorithms. This procedure is known as feature extraction. It involves converting unprocessed data into a more comprehensible and useful representation, and it is an essential stage in machine learning and data analysis [33]. It seeks to locate and extract from raw data the most pertinent and non-redundant characteristics, therefore improving the efficiency of machine learning algorithms. There are many techniques for feature extraction in text data. However, a combination of bag-of-words and TF-IDF (Term Frequency-Inverse Document Frequency) were the chosen methods.

##### Bag-of-Words (BoW)

The bag-of-words model is a method of representing text as an unordered collection of words. It is commonly used in natural language processing (NLP) and information retrieval (IR). The bag-of-words model disregards grammar and word order, but it keeps multiplicity. This means that if a word occurs multiple times in a document, it is counted multiple times in the bag-of-words representation of that document [34].

##### Term Frequency (TF)

A statistical metric called Term Frequency-Inverse Document Frequency (TF-IDF) assesses a word’s significance in a document within a set of documents [35]. It is a commonly used method in text mining and information retrieval.

The number of times a term (word) appears in a document is its term frequency (TF). It is a straightforward indicator of the frequency with which a term appears in a given document.

##### Inverse Document Frequency (IDF)

The measure of a term’s frequency of occurrence in a set of documents is called Inverse Document Frequency (IDF). It is calculated by taking the logarithm of the total number of documents and dividing it by the number of documents that contain the term.

##### TF-IDF

The result of TF and IDF is TF-IDF. It evaluates a term’s significance in a document by considering both its occurrence in the document and its rarity within the collection. A term’s importance to the document and not merely its commonness is indicated by a term’s high TF-IDF score [35].

### 3.3. Artificial Intelligence Algorithms

1. “lbfgs” Logistic Regression

“lbfgs” logistic regression refers to a specific type of logistic regression algorithm that uses the limited-memory Broyden–Fletcher–Goldfarb–Shanno (L-BFGS) optimization method. Logistic regression is a statistical method used for binary classification problems. Based on one or more predictor factors, it estimates the probability of a binary result (1/0, Yes/No, or True/False). L-BFGS is a popular optimization algorithm used for finding the minimum of a function, typically in the context of machine learning and numerical optimization. It is an iterative method that belongs to the family of quasi-Newton methods. L-BFGS is known for being memory-efficient and suitable for problems with a large number of parameters.

When combined, “lbfgs Logistic Regression” suggests that logistic regression is being used with the L-BFGS optimization method to train a binary classification model. This combination is often employed when dealing with machine learning tasks that require optimizing the logistic regression model’s parameters to fit the data.

The sigmoid function, a logistic function, is used in logistic regression to map predictions and their probability. An S-shaped curve known as the sigmoid function transforms any real number into a range between 0 and 1.

For logistic regression, the sigmoid function is known as an activation function and is described as follows:

(1) $$P\left(Y=1\right|X)=\frac{1}{1+e^{-\left(\beta 0+\beta 1X1+\beta 2X2+\cdots +\beta nXn\right)}}$$

where *P*(*Y* = 1∣*X*) is the probability of the target variable *Y* being 1 given the predictors *X*, “*β*_0_, *β*_1_, *β*_2_, …, *β_n_*” are the coefficients of the model, “*X*_1_, *X*_2_, …, *X_n_*” are the predictor variables, and *e* is the base of the natural logarithm (Euler’s number).

The logistic function $\frac{1}{1+e^{-x}}$ transforms the output of a linear equation (*β*_0_ + *β*_1_ *X*_1_ + *β*_2_ *X*_2_ + … + *β*_n_ *X*_n_) into a range between 0 and 1, representing probabilities [36].

The coefficients *β*_0_, *β*_1_, *β*_2_, …, *β_n_* are estimated using optimization algorithms (often maximum likelihood estimation) to minimize the error between predicted probabilities and the actual outcomes in the training data.

2. “liblinear” Logistic Regression

liblinear is a library for large linear classification, which includes logistic regression. It is a popular choice for training logistic regression models on large datasets because it is fast and efficient. Coordinate descent is the method that liblinear utilizes to solve the logistic regression optimization issue.

Coordinate descent is an iterative algorithm that works by optimizing one coordinate at a time. It is a simple and efficient algorithm, and it is well suited for large datasets. The “liblinear” solver is an optimization algorithm used to fit the logistic regression model. It is based on a linear support vector machine (SVM) algorithm and works well for small- to medium-sized datasets. It optimizes the logistic regression cost function using techniques like coordinate descent. Refer to Equations (1) and (2) for reference.

3. “newton-cg” Logistic Regression

The “newton-cg” solver stands for “Newton Conjugate-Gradient”. It is a numerical optimization technique that combines the Newton–Raphson method with the conjugate gradient method to determine the best logistic regression model parameters. This solver is known for its efficiency and suitability for a wide range of logistic regression problems.

When combined, “newton-cg Logistic Regression” indicates that logistic regression is being used, and the “newton-cg” solver is chosen as the optimization method to train the logistic regression model. This combination is often applied when you have a logistic regression problem that benefits from the characteristics of the “newton-cg” optimization algorithm. Refer to Equations (1) and (2) for reference.

4. “sag” Logistic Regression

Stochastic average gradient descent (SAG) logistic regression is a solver that uses stochastic average gradient descent to train a logistic regression model. Stochastic average gradient descent (SAG) is an iterative algorithm that updates the model parameters by averaging the gradients of a small batch of samples at each iteration. SAG is a fast and efficient algorithm for training logistic regression models on large datasets. It is particularly well-suited for datasets with a large number of features. SAG is also less sensitive to the initial parameter values than other logistic regression solvers. Refer to Equations (1) and (2) for reference.

5. Random Forest

Random Forest is a supervised machine learning approach that can be used for regression and classification applications. In order to generate a final forecast, it builds a large number of decision trees during the training phase and averages them.

The foundation of Random Forests is the idea of ensemble learning, which combines the predictions of several different models to generate a more accurate forecast. Because of this, Random Forests are less likely than individual decision trees to overfit.

In contrast to decision tree classifiers, which receive the root node by using the Gini index or information gain, Random Forests obtain the root node randomly, and the splitting of the attribute nodes also occurs randomly [37].

(2) $$\begin{array}{c} Gini\;Index=1-\sum_{i=1}^{n}{\left(P_{i}\right)}^{2}\\ =1-[{\left(P_{+}\right)}^{2}+{\left(P_{-}\right)}^{2}] \end{array}$$

Here, P_+_ represents the likelihood of a positive class, whereas P_−_ denotes the likelihood of a negative class [38].

6. Perceptron

Its predictions are based on a linear predictor function, which combines the feature vector with a set of weights because it is a linear classifier.

A collection of input values is fed into the perceptron, and it multiplies the results by a set of weights. After that, an activation function is applied to the total of these products to provide an output. Usually a step function, the activation function determines whether the input is higher or less than a threshold and outputs a binary value (0 or 1) accordingly. Many algorithms can be used to train a perceptron; however, the perceptron learning algorithm is the most widely used one. In order for this technique to correctly identify all of the training data, iteratively modifying the perceptron’s weights is how it operates.

The following is the fundamental equation for a perceptron’s operation:

(3) $$f\left(x\right)=\left\{\begin{array}{c} 1\;\;\;\;if\;w\cdot x+b>0\\ 0\;\;\;otherwise \end{array}\right.$$

where w is a vector of weights with actual values, b is bias, which is an independent component that modifies the boundary away from the origin, and x is the input x values’ vector.

(4) $$\sum_{i=1}^{m}w_{i}x_{i}$$

Here, m denotes the number of perceptron inputs.

Either “1” or “0” can be used to represent the output. Depending on the activation function being utilized, it can also be represented as “1” or “−1” [39].

7. Ridge Classifier

A Ridge Classifier is a linear algorithm for classification that has the Ridge regression algorithm as its foundation. Based on features, it is used to categorize data into two or more classes. Ridge regression is a regularized linear regression approach that prevents overfitting by including a penalty term in the loss function. Large model coefficients are penalized by this regularization term, which makes the model learn fewer complex correlations between the target variable and the data.

The Ridge Classifier works by first converting the target variable into {−1, 1} and then treating the problem as a regression task. It then uses the Ridge regression algorithm to learn a linear model that can predict the target variable. The predicted class is then determined by the sign of the predicted target value.

The Ridge Classifier’s objective function aims to minimize the following cost function:

(5) $$\mathrm{Cos}t=\sum_{i=1}^{n}\left(loss\;(y_{i}{,\;y}_{i}\right){+\alpha \left|\left|w\right|\right|}^{2})$$

Here, loss $\left(y_{i}{,\;y}_{i}\right)$ represents the loss function used for classification (e.g., typically logistic loss or squared hinge loss), *α* is the regularization parameter that controls the strength of the penalty term, and w denotes the coefficients (weights) of the linear function.

The Ridge Classifier seeks to find the optimal weights (w) that minimize this cost function, balancing between fitting the training data well and keeping the coefficients small to prevent overfitting.

8. CatBoost Classifier (CatBoost)

A CatBoost classifier is a gradient boosting classifier made especially to deal with features that fall into one of the categories. It employs a range of methods to enhance gradient boosting’s effectiveness on categorical data, such as the following:

- Ordered boosting: CatBoost makes use of ordered boosting to discover connections among categorical variables with an inherent ordering.
- Feature hashing: To effectively handle a high number of categorical features, CatBoost takes advantage of feature hashing.
- Oblivious trees: CatBoost makes use of oblivious trees, which are decision trees that do not care which way the category features are arranged.

(6) $$F(x)=B_{0}+\sum_{i=1}^{n}\cdot f_{m}(x)$$

Here, *F*(*x*) is the final prediction for input *x*, and *B*_0_ is the initial prediction (often a global constant or the average of the target variable). *M* is the number of trees in the ensemble, and *f_m_*(*x*) represents the prediction of the *m*-th tree for input *x*.

To arrive at the end prediction, the starting prediction is added to the increments predicted by each individual tree *f_m_*(*x*). These trees are built using the CatBoost algorithm step by step, with each tree built trying to rectify the mistakes the ensemble has made thus far.

9. Nearest Centroid Classifier

A Nearest Centroid Classifier is a simple and effective classification algorithm that assigns a new data point to the class whose centroid is closest to the new data point. The centroid of a class is the mean of all the data points in that class.

To classify a new data point using the Nearest Centroid Classifier, the following steps are typically taken:

- Determine how far the new data point is from each class’s centroid.
- Assign the newly discovered data point to the class whose centroid is nearest to it.

Being a non-parametric technique, the Nearest Centroid Classifier does not make any assumptions about the data’s underlying distribution. Because of this, the Nearest Centroid Classifier is an adaptable method that works well for a range of applications.

Let us define the key components:

- *x_ij_*: Feature vector of the *i*-th sample in class *j*.
- *C_j_*: Centroid for class *j*.
- *N*: Number of features.
- *n_j_*: Number of samples in class *j*.

The steps involved are detailed below.

#### 3.3.1. Centroid Calculation

For each class *j*, calculate the centroid *c_j_* as the mean of the feature vectors belonging to that class:

(7) $$C_{j}=\frac{1}{n_{j}}\sum_{i=1}^{n_{j}}\cdot x_{ij}$$

This equation computes the centroid *c_j_* for class *j* by averaging the feature vectors x_ij_ within that class.

#### 3.3.2. Distance Calculation 

Given a new test instance x_test_, compute the distance from x_test_ to each centroid *c_j_* using a distance metric like Euclidean distance:

(8) $$\mathrm{Distance}(x_{\mathrm{test}},\;c_{j})=\sqrt{\sum_{i=1}^{N}}\cdot {\left({x^{i}}_{\mathrm{test}}-{c_{j}}^{i}\right)}^{2}$$

Equation (9) calculates the Euclidean distance between the test instance x_test_ and each centroid *c_j_* across all features.

10. Stochastic Gradient Descent (SGD Classifier)

Stochastic Gradient Descent is a training algorithm for machine learning models that uses optimization. It updates the model parameters iteratively in the direction of the loss function’s negative gradient. The direction that the parameters should be adjusted in order to minimize the loss is indicated by the gradient of the loss function.

Because SGD is a stochastic algorithm, it only uses one training example at a time to update the model’s parameters. For training huge datasets, where it would be impossible to change the parameters using the full dataset at once, this makes SGD particularly effective [40].

*Y* = *mx* + *b* (9)

Equation (10) is the straight-line equation where m is the slope and b is its intercept while *m* = *m* − *əm* and *b* = *b* − *əb*. These are parameters with a small change *Cost* = $\frac{1}{N}\sum_{i=1}^{N}\left(Yi’-Yi\right)$^2^; this is the cost function for N samples.

11. Support Vector Classifier—SVC (kernel = “linear”, C = 0.025)

SVC is a linear kernel support vector machine (SVM) classifier with a 0.025 regularization parameter. SVMs are a class of machine learning algorithms that are applicable to applications involving both regression and classification. In order to divide the data points into two classes, they locate a hyperplane in the feature space.

The linear kernel is a simple kernel that calculates the dot product between two data points. This makes it a good choice for datasets with a small number of features. The regularization parameter controls how much the model is penalized for misclassifying data points. A model with a larger regularization parameter will be less likely to overfit the training set and be more complex [40]. The Support Vector Classifier (SVC) is a specific implementation of the support vector machine (SVM) algorithm used for classification tasks. The mathematical representation of SVC closely aligns with the general formulation of SVMs for binary classification. For simplicity, let us consider a linear SVC for linearly separable classes. Given the training data (x_i_, y_i_) where x_i_ represents feature vectors and y_i_ represents class labels (+1 or −1 for binary classification), the following is determined:

The decision function for SVC is similar to the SVM and is represented as follows:

(10) $$f\left(x\right)=sign\left(w\cdot x+b\right)$$

where x represents the input feature vector, w represents the weight vector, *b* is the bias term, and ⋅ denotes the dot product between vectors.

The optimization problem for SVC aims to find the optimal hyperplane that separates the classes while maximizing the margin and minimizing classification errors. It can be represented as follows:

(11) $$\begin{array}{c} \mathrm{minimize}=(\frac{1}{2}{\left||w|\right|}^{2})\\ \mathrm{subject}\;\mathrm{to}\;y_{i}(w\cdot x_{i}+b)\geq 1\;\mathrm{for}\;i=1,2,\ldots ,n \end{array}$$

where ||w|| represents the Euclidean norm or w, and the objective function minimizes $\left|\left|w\right|\right|$^2^ to maximize the margin.

The constraints ensure that data points are correctly classified and are sufficiently far from the decision boundary (at a distance of at least 1/||w||).

12. Support Vector Classifier—SVC (kernel = “rbf”, gamma = 2, C = 1)

The Support Vector Classifier is a support vector machine (SVM) classifier with a regularization parameter of 1 and a radial basis function (RBF) kernel. One kind of machine learning method that may be applied to both classification and regression problems is RBF SVMs. In order to divide the data points into two classes, they locate a hyperplane in the feature space.

A non-linear kernel called the RBF kernel uses the distance between two data points to determine how similar they are. For datasets where the data are not linearly separable, this makes it a good option. The amount that the model is penalized for incorrectly identifying data points is determined by the regularization parameter. A model with a larger regularization parameter will be more sophisticated and less prone to overfit the training set. Refer to Equations (11) and (12) for reference.

13. LinearSVC

LinearSVC is a support vector machine (SVM) classifier with a linear kernel. SVMs are a class of machine learning algorithms that are applicable to applications involving both regression and classification. In order to divide the data points into two classes, they locate a hyperplane in the feature space. LinearSVC is similar to the linear SVM and SVC but is implemented with a different optimization algorithm, typically based on the LIBLINEAR library, making it more suitable for large-scale datasets. The optimization problem is expressed below.

LinearSVC is a faster and more efficient implementation of SVM classification for the case of a linear kernel. It also has fewer parameters to tune, making it easier to use.

(12) $$minimize=(\frac{1}{2}||w||2+C\sum_{i=1}^{n}max(0,1-yi(w\cdot xi+b)))$$

Here, w represents the weight vector, *b* is the bias term, *C* is the regularization parameter, controlling the trade-off between maximizing the margin and minimizing the classification error, and ⋅ denotes the dot product between vectors.

The objective function consists of two terms: the first term minimizes the norm of the weight vector to maximize the margin, while the second term represents the hinge loss, penalizing misclassifications. The parameter *C* balances the importance of these two terms, controlling the regularization strength.

LinearSVC aims to find the optimal w and *b* that define the hyperplane separating the classes by solving this optimization problem using efficient algorithms. It constructs a linear decision boundary in the input space to classify data points into different classes. LinearSVC is particularly efficient for large-scale datasets and linearly separable problems, offering a computationally tractable solution for linear classification tasks.

14. ZeroR Classifier

The ZeroR classifier is among the most straightforward and fundamental machine learning algorithms. It is essentially a baseline or reference model that functions as a straightforward benchmark for assessing the effectiveness of more intricate machine learning models rather than a learning algorithm in the conventional sense.

The training dataset’s most frequent class or value is the only factor used by the ZeroR algorithm to generate predictions. It allocates each instance in a classification task to the class that occurs most frequently in the training set. In regression tasks, each occurrence is given a constant value, usually the target variable’s mean or median. 

The principle behind ZeroR is straightforward. For classification tasks, it predicts the most frequent class label from the training data for all instances in the test data, disregarding any features or input variables. Essentially, it always predicts the same class, the mode of the target variable in the training set.

For regression tasks, it predicts the mean or median of the target variable in the training set for all instances in the test data, irrespective of the input features.

15. Decision Tree Classifier

A decision tree classifier is an algorithm for supervised machine learning that can be applied to applications involving regression and classification. It functions by building a model of the data that resembles a tree, with each node in the tree representing a decision. After that, the model makes predictions on fresh data points using this tree [41].

(13) $$E(S)=\sum_{i=1}^{c}-p_{1}\log_{2}p_{1}$$

Here, *p* is the *i*-th order probability.

(14) $$G(S,\;C)=E(S)-\sum_{w2\mathrm{values}(C)}\frac{Sw}{S}\;E(S_{W})$$

16. Passive Aggressive Classifier

This classifier is particularly useful when dealing with large-scale, streaming, or online learning scenarios where data arrive sequentially, and models need to adapt to changes. The “passive-aggressive” name is derived from the algorithm’s behavior when making updates to its model. It adjusts its model parameters based on a trade-off between making a “passive” update (minimizing the loss function) and an “aggressive” update (correcting misclassifications).

The update rule for the Passive Aggressive Classifier is typically based on the loss function and the gradient of the loss.

Given *w* as the weight vector, *x* as the input vector, *y* as the true label, ŷ as the predicted label, *η* as the learning rate, and *C* as the regularization parameter, the following is determined:

The update rule for the weight vector *w* of the Passive Aggressive Classifier is often represented as follows:

(15) $$W=w+\eta \cdot (y-ŷ+\frac{\partial L}{\partial w})x-\eta \cdot C\cdot w$$

where *y* − ŷ represents the loss or error term, $\frac{\partial L}{\partial w}$ represents the gradient of the loss with respect to the weight vector, and the regularization term *C*⋅*w* penalizes large weights to control overfitting.

The Passive Aggressive algorithm adjusts the weights in such a way that it tries to minimize the loss while staying close to the previous weight vector. The aggressiveness of the update is controlled by the learning rate (*η*) and the magnitude of the error term.

17. Extra Tree Classifier

The Extra Tree Classifier is an ensemble learning algorithm that predicts using a set of decision trees. While it is comparable to the Random Forest Classifier, there are a few significant differences. The Extra Tree Classifier splits the features and samples randomly at each node of the tree, while the Random Forest Classifier uses a more informed approach to selecting features and samples. The Extra Tree Classifier does not bootstrap the training data, while the Random Forest Classifier does.

18. Random Patches

Random Patches is an ensemble machine learning method that blends the ideas of random subspaces and bagging. Its main application is to enhance the functionality of machine learning models—particularly Random Forests and decision trees. Because Random Patches focuses on randomizing both the data and the features used for training, it is also known as “Feature Bagging”.

#### 3.3.3. Sampling Data

For each model *i* in the ensemble *M*, a subset of the training data *D_i_* is randomly sampled with replacement from the original training set *D*. This sampling might include *N* samples from *D*, where *N* is less than the total number of samples in *D*.

#### 3.3.4. Selecting Features

For each subset *D_i_*, a random subset of features *F_i_* is chosen. This might involve selecting a certain number *K* of features randomly from the total feature set *F*.

These randomly generated subsets are then used to train other models (such as decision trees or other classifiers) using the Random Patches technique. The predictions from each model may be combined by voting (for classification) or averaging (for regression) during inference (when producing predictions) to produce the final forecast.

19. Voting Classifier

A voting classifier is an ensemble learning algorithm that creates predictions by combining several classifiers. In order to arrive at a final prediction, a set of base classifiers is trained using the training data. The base classifiers’ predictions are then averaged.

The voting classifier combines the predictions from multiple base models *M*1, *M*2, …, *Mn* as follows:

For Hard Voting,

(16) $$\mathrm{Final}\;\mathrm{Prediction}=\mathrm{argmax}(\sum_{i=1}^{n}1(M_{i}(x)=c))$$

where 1 is the indicator function, *M_i_*(x) represents the prediction of the *i*th model for input x, and *c* is the class label.

For Soft Voting (for classifiers that output probabilities),

$$\mathrm{Final}\;\mathrm{Prediction}=\mathrm{argmax}(\sum_{i=1}^{n}P(M_{i}(x)=c))$$

where *P*(*M_i_*(x) = *c*) represents the probability of class *c* predicted by the *i*th model for input x.

20. Stacked Generalization

Super learning, another name for stacked generalization, is an ensemble learning method that combines the predictions of several machine learning models to generate a final prediction that is more accurate. The way it operates is by using the underlying models’ outputs to train a meta-model, also called a stacking model. In order to minimize the total error, the meta-model learns how to combine the predictions of the basic models. 

Stacked generalization (stacking) involves combining the predictions from multiple base models to train a meta-model that learns to make the final predictions. Let us break down the mathematical representation step by step.

Given the training dataset (*X*, *y*) and *M* diverse base models denoted as *f*_1_, *f*_2_, …, *f_M_*, the following is determined:

Base Model Training: Train the base models *f_i_* on the training dataset *X* to obtain predictions:

ŷ*_i_* = *f_i_*(*X*), for *i* = 1, 2, …, *M*

Formation of the Meta-Features: Use the predictions of these base models as new features (meta-features) for the meta-model. Form a new dataset consisting of the predictions:

*X*_meta_ = [ŷ_1_, ŷ_2_, …, ŷ*_M_*]

Here, *X*_meta_ is a matrix where each row represents an instance in the original dataset, and each column represents the predictions made by one of the base models.

Training the Meta-Model: Train a meta-model (meta-learner) *g* using the meta-features *X*_meta_ and the true target values *y*:

*g*(*X*_meta_) = *y*   multilayer perceptron classifier

is a kind of artificial neural network (ANN) that is suitable for jobs requiring both regression and classification. Multiple layers of networked nodes, or neurons, comprise MLPs. Every neuron in one layer is linked to every other neuron in the layer above it.

Backpropagation is one type of supervised learning technique used to train MLP classifiers. The technique of backpropagation involves minimizing the loss function by modifying the weights of the connections between neurons. The MLP’s prediction accuracy of the target values is indicated by the loss function.

Let us consider a simple MLP classifier with multiple hidden layers. *X* represents the input features, *W*^(*i*)^ represents the weights for the connections between the *i*th layer and the (*i* + 1)th layer, *b*^(*i*)^ represents the bias terms for the *i*th layer, and *A*^(*i*)^ represents the activation of the *i*th layer.

The forward pass through an MLP with multiple hidden layers can be represented mathematically as follows:

Input Layer to Hidden Layer (Layer 1 to Layer 2):

*Z*^(1)^ = *X*·*W*^(1)^ + *b*^(1)^
*A*^(1)^ = σ(*Z*^(1)^) (17)

where *Z*^(1)^ is the weighted sum of the inputs, *A*^(1)^ is the activation of the first hidden layer, and *σ* is the activation function (e.g., ReLU, sigmoid, or tanh) applied element-wise to *Z*^(1)^.

Hidden Layers (Layer 2 to Layer N − 1): For each subsequent hidden layer *i* (from 2 to N − 1),

*Z*^(*i*)^ = *A*^(*i*−1)^·*W*^(*i*)^ + *b*^(*i*)^
*A*^(*i*)^ = σ(*Z*^(*i*)^) (18)

where *Z*^(*i*)^ is the weighted sum of activations from the previous layer, and *A*^(*i*)^ is the activation of the *i*th hidden layer.

Last Hidden Layer to Output Layer (Layer N − 1 to Output Layer):

*Z*^(*N*)^ = *A*^(*N*−1)^·*W*^(*N*)^ + *b*^(*N*)^
ŷ = σ(*Z*^(*N*)^) (19)

where *Z*^(*N*)^ is the weighted sum of activations from the last hidden layer, and ŷ is the final output or prediction.

21. Bernoulli Restricted Boltzmann Machine (Bernoulli RBM)

A Bernoulli Restricted Boltzmann Machine is a kind of neural network model, more precisely an artificial neural network with generative stochastic properties. RBMs are employed in collaborative filtering, dimensionality reduction, and feature learning, among other unsupervised learning applications. The probability distribution type utilized for the binary units in the model is indicated by the term “Bernoulli” in the name.

Two levels of units make up a Bernoulli RBM: a visible layer and a concealed layer. The input data are represented by the visible layer, and the latent representation of the data is represented by the hidden layer. Because they are binary, the units in the visible layer can either be on or off. The concealed layer’s units are binary as well.

It has a mathematical representation in terms of probability related to the visible and hidden units and the energy function.

Supposing that we have a Bernoulli RBM with M hidden and N visible units, the following is determined:

Energy Function: The energy of a configuration of visible and hidden units in an RBM is given by the following:

(20) $$\;E(v,h)=\sum_{i=i}^{N}\cdot \sum_{j=1}^{M}\cdot \;W_{\mathrm{ij}}v_{i}h_{j}-\sum_{i=i}^{N}\cdot a_{i}v_{i}-\sum_{j=1}^{M}\cdot b_{j}h_{j}$$

where V = (*v*_1_, *v*_2_, …, *v_N_*) represents the states of visible units, h = (*h*_1_, *h*_2_, …, *h_M_*) represents the states of hidden units, *W_ij_* are the weights between visible unit *i* and hidden unit *j*, and *a_i_* and *b_j_* are the biases for visible unit *i* and hidden unit *j*, respectively.

Joint Probability: The joint probability of a configuration of visible and hidden units in an RBM is defined using the energy function:

(21) $$P(v,h)=\frac{1}{z}\;e^{-E(v,h)}$$

where *Z* is the normalization constant (partition function) calculated by summing over all possible configurations of visible and hidden units:

(22) $$Z=\sum_{v}\cdot \;\sum_{h}\cdot e^{-E(v,h)}$$

Conditional Probabilities: The conditional probabilities for the states of the hidden units given the visible units and vice versa in a Bernoulli RBM are as follows:

(23) $$P(\mathrm{hj}=1|v)=\sigma (\sum_{j=1}^{N}\cdot W_{\mathrm{ij}}v_{i}+b_{j})$$

(24) $$P(v_{i}=1|h)=\sigma (\sum_{j=1}^{M}\cdot W_{\mathrm{ij}}h_{j}+a_{i})$$

(25) $$\;\mathrm{where}\;\sigma (x)\;\mathrm{is}\;\mathrm{the}\;\mathrm{sigmoid}\;\mathrm{function}:\;\sigma (x)=\frac{1}{1+e^{-x1}}$$

In order to train a Bernoulli RBM, one usually maximizes the log-likelihood of the training data in order to determine the weights and biases. To update the parameters based on observed data samples, methods like Stochastic Gradient Descent and Contrastive Divergence (CD) are frequently employed.

22. AdaBoost Classifier

An AdaBoost Classifier is an ensemble learning approach that builds a strong classifier by combining several weak classifiers. It trains a set of weak classifiers iteratively using the training data and then modifies the weights of the weak classifiers according to how well they perform. A weighted average of the predictions made by the weak classifiers makes up the AdaBoost Classifier’s final prediction.

The ensemble prediction is computed by combining the weighted predictions of all the weak learners:

(26) $$H(x)=\mathrm{sign}(\sum_{t=1}^{T}\cdot \alpha_{t}\;h_{t}\;(x))$$

where *H*(*x*) is the final prediction for sample *x*, *α_t_* is the weight of weak learner *h_t_*, and *T* is the total number of weak learners.

23. Gradient Boosting Classifier

A gradient boosting classifier is an ensemble machine learning algorithm used for classification tasks. It builds predictive models by combining multiple decision trees through gradient descent optimization. This method is known for its high predictive accuracy and robustness in handling complex relationships in data. It is widely used in applications like spam detection, fraud detection, and image classification. Key parameters to tune include the learning rate, the number of trees, and tree depth.

(27) $$F(x)=\sum_{t=1}^{T}\cdot \mathrm{learning\_rate}\times {\mathrm{weak\_learner\_prediction}}_{t}$$

Gradient boosting adds weak learners one after the other that outperform the prior models in order to optimize the ensemble model. An effective prediction model is produced when each new learner fixes the mistakes committed by the previous ensemble.

24. Ordinal Learning Model

One kind of machine learning model that is used to predict ordered categorical variables is called an ordinal learning model. This indicates that rather than just predicting the class label, the model also predicts the rank or degree of a variable. When data are organically arranged, such as in customer satisfaction surveys or medical diagnoses, ordinal learning models are frequently employed.

Because conventional classification and regression models do not account for the ordered character of the target variable, they are not immediately appropriate for ordinal data. To solve this problem and produce predictions that take into account the data’s ordinal structure, ordinal learning models were created.

Ordinal Logistic Regression is an illustration of an Ordinal Regression model. The cumulative probabilities connected to the ordered categories provide the formula for Ordinal Logistic Regression. Take variable Y, for example, which has K-ordered categories (low, medium, and high).

The cumulative probabilities for a given category *k* are represented as follows:

(28) $$P\left(Y\leq k\right|X)=\frac{1}{1+e^{-\left(\alpha k+\beta 1X1+\beta 2X2+\cdots +\beta pXp\right)}}$$

where *P*(*Y* ≤ *k*∣*X*) is the probability that the outcome *Y* is less than or equal to category *k* given the input features *X*, *αk* is the intercept specific to category *k*, and *β*_1_, *β*_2_, …, *β_p_* are the coefficients associated with each feature *X*_1_, *X*_2_, …, *X_p_*, respectively.

This formula extends logistic regression to accommodate ordered categorical outcomes. It is based on the logistic function, also known as the sigmoid function. Depending on the input features, the parameters αk and β are determined during training to maximize the probability of detecting the provided ordinal outcomes.

25. Extreme Gradient Boosting (XGBoost)

Extreme gradient boosting is an ensemble learning algorithm, which means that it creates a stronger prediction by combining the predictions of several weak learners. Decision trees are the weak learners in XGBoost. The decision trees are constructed successively by XGBoost, and each tree is trained to fix the mistakes of the one before it. An objective function is made up of a regularization term, and a loss function is optimized by XGBoost. The loss function L and the regularization term Ω add up to the objective function that has to be minimized:

Objective = *L*(predictions, labels) + Ω(model) (29)

The particular equations utilized in the execution of XGBoost include the optimization of the objective function, which combines regularization and the loss function, as well as the gradient and Hessian computations for building and refining the ensemble of trees.

26. Decision Stump

A decision stump is a straightforward machine learning model made up of a decision tree with only one level. The algorithm for binary classification relies on the value of a solitary input feature to generate predictions. Decision stumps are frequently employed as building blocks in more intricate machine learning methods, including gradient boosting machines and AdaBoost.

Finding the optimal split for the data based on a single feature is the formula for a decision stump. Assume that we have a dataset that contains the target variable y and one feature, x.

The decision stump’s split condition can be represented as follows:

If *x* < *θ*:       predict class *c*1 Else:       predict class *c*2

where *x* is the feature value, *θ* is the threshold value used to split the data, and *C*_1_ and *C*_2_ are the predicted classes on either side of the split.

The decision stump algorithm looks over the feature space to identify the optimal threshold value that reduces a given criterion, which is frequently information gain or purity. For example, it could maximize Gini impurity or minimize misclassification errors in classification tasks.

27. Complement Naïve Bayes (CNB)

Complement Naïve Bayes is a Naive Bayes classifier variation created to overcome some of the drawbacks of the original Naive Bayes algorithm.

The Naive Bayes classifier’s primary drawback is its assumption that the features are unrelated to one another. In real-world data, this is frequently not the case because features might be connected. By identifying the relationships between characteristics and applying this knowledge to increase classification accuracy, Complement NB overcomes this drawback.

The Naive Bayes classifier’s tendency to favor the majority class in unbalanced datasets is another drawback. Utilizing a weighted log-likelihood function that assigns greater weight to the minority class, Complement NB overcomes this constraint.

Bayes’ theorem provides the following formula for the conditional probability in Complement Naive Bayes:

(30) $$P(c|x)=\frac{P(x|c)\cdot P(c)}{P(x)}$$

where *P*(*c*∣x) is the probability of class *c* given the features x, *P*(x∣*c*) is the likelihood of observing the features x given class *c*, *P*(*c*) is the prior probability of class *c*, and *P*(x) is the evidence probability.

The class-conditional probability is computed differently in Complement Naive Bayes. Rather than modeling the chance of observing features given the class directly, CNB determines the chance of the features given the class’s absence (i.e., the complement of the class):

*P*(x∣¬ *c*)

28. Multinomial Naïve Bayes (MNB)

Multinomial feature classification challenges are the specialty of MNB. Features that can have a set number of values, such as the frequency with which a word appears in a document, are known as multinomial features.

The way MNB operates is by figuring out how likely each class is given the characteristics of the data item. Next, it is predicted that the class with the highest probability is the right class.

*P*(x∣*c*) represents the likelihood of observing the features x given class *c*.

Assuming feature independence, Multinomial Naive Bayes estimates this likelihood as the product of the probabilities of each feature (word or token) given the class.

(31) $$P(x|c)=\prod_{i=1}^{n}\cdot P(x_{i}|c)$$

Here, *i* represents the *i*-th feature (word or token), and *n* is the total number of features.

### 3.4. Justification for the Choice of Ensemble Models/Techniques Chosen

Twelve ensemble models—which include Random Forest, CatBoost, Random Patches, voting, stacked generalization, multilayer perceptron (MLP), Bernoulli RBM, AdaBoost, gradient boosting, ordinal learning model (OLM), XGBoost, and Extra Tree—were selected from a total of 29 methods. The fact that all 12 algorithms made their decisions by using one or more ensemble techniques (such as stacking, voting, bagging, and boosting) should be noted. Since AdaBoost, bagging, and Random Forest are existing meta estimators, the algorithms in the ensemble were selected to offer a fresh combination of techniques. A combination of Random Forest, SVC, and decision tree using the voting ensemble technique or Random Forest, SVC, and gradient boosting using the stacking ensemble technique was seen to have produced superior outcomes, especially when compared to traditional algorithms.

### 3.5. Performance Matric for Evaluation

The effectiveness of the supervised artificial intelligence algorithms on the test data was evaluated using a confusion matrix. It is particularly useful for evaluating a model’s ability to predict different classes or categories within a dataset. A confusion matrix is used to show the outcomes of a model’s attempt to classify a batch of data points into one of several predefined classes or labels. In a typical confusion matrix, the rows represent the actual classes, or ground truth, and the columns represent the classes that the model predicted. The cells of the matrix include counts of the number of data points that belong to each combination of actual and predicted classes. A confusion matrix can yield the following four crucial values:

- True Positives (TPs): In these instances, the model successfully predicted the positive class and identified the positive class (the class it truly belongs to). This example shows bogus news that was labeled as such.
- True Negatives (TNs): In these cases, the model properly identified the negative class as not falling under the positive category. This instance indicates real news that was classified as real news.
- False Positives (FPs): This happens when the model predicts the positive class when it should have predicted the negative class, but it does so wrongly. This example shows that genuine news was mislabeled as false news.
- False Negatives (FNs): This happens when a model predicts a negative class mistakenly when it should have predicted a positive class. In this case, bogus news was mistakenly identified as authentic news. All these metrics are summarized in Table 2.

These values can be used to produce a number of performance measures, including the following:

- Accuracy: It assesses the overall accuracy of the model’s predictions.

(32) $$Accuracy=\frac{\left(TP+TN\right)}{\left(TP+TN+FP+FN\right)}$$

- Precision: It assesses how well the model predicted outcomes that materialized.

(33) $$Precision=\frac{TP}{\left(TP+FP\right)}$$

- Recall (Sensitivity or True Positive Rate): It evaluates how well the model detects every instance of positivity.

(34) $$Recall=\frac{TP}{\left(TP+FN\right)}$$

- Specificity (True Negative Rate): It evaluates the model’s ability to identify every instance of negativity.

(35) $$Specificity=\frac{TN}{\left(TN+FP\right)}$$

- F1 Score: It provides an equitable evaluation of a model’s efficacy and is the harmonic mean of recall and precision.

(36) $$F1\;Score=2\times \frac{\left(Precision\times Recall\right)}{\left(Precision+Recall\right)}$$

- Matthews Correlation Coefficient (MCC): This is employed to assess the performance of a binary classification model. It offers a fair measure by taking into consideration false positives, false negatives, true positives, and true negatives—even in situations when the classes have different sizes. The MCC is ascertained using the following formula:

(37) $$MCC=2\times \frac{TP\times TN-FP\times FN}{\sqrt{\left(TP+FP\right)}\left(TP+FN\right)\left(TN+FP\right)\left(TN+FN\right)}$$

- KAPPA: Also referred to as Cohen’s Kappa, this is a statistic used to assess how well a classification model is doing, particularly when the distribution of the classes is unbalanced. It assesses the degree of agreement between the actual and anticipated categories, accounting for the likelihood that agreement could also occur.

(38) $$KAPPA=\frac{P_{o}-P_{e}}{1-P_{e}}$$

Here, P_o_ is the observed agreement, the ratio of instances that were correctly predicted by the model, and P_e_ is the expected agreement, the probability that the model’s predictions and the true labels would agree by chance. 

The observed agreement (P_o_) is computed by dividing the total number of instances by the sum of the diagonal members of the confusion matrix.

The product of the marginal probabilities of the true and predicted labels, added together for each class, is the expected agreement (P_e_).

- Area Under the Receiver Operating Characteristic curve (AUC-ROC or simply AUC):

This is a performance metric that is commonly used to address binary classification problems. The ROC curve visually illustrates the trade-off between the true positive rate (sensitivity) and the false positive rate (specificity) for different categorization model thresholds.

The area under this ROC curve, or AUC, is a single scalar statistic that summarizes the model’s performance over a variety of classification criteria. A higher AUC usually indicates better discrimination between positive and negative cases.

- False Discovery Rate (FDR): This is a statistical metric applied to hypothesis testing and binary classification. It shows the percentage of false positives, or inaccurate positive predictions, among all of a model’s positive predictions. The expected ratio of false positives to all positive test results is known as the false positive ratio, or FDR, in the context of hypothesis testing.

The False Discovery Rate is calculated using the following formula:

(39) $$FDR=\frac{FP}{FP+TP}$$

- False Negative Rate (FNR): Sometimes referred to as the Miss Rate, this is a binary classification metric that quantifies the percentage of actual positive cases that a model mistakenly predicts as negative. It has the following definition:

(40) $$FNR=\frac{FN}{FN+TP}$$

- False Positive Rate (FPR): Often called the False Alarm Rate or Fall-Out, this binary classification indicator measures the proportion of real negative events that a model incorrectly interprets as positive. It is defined as follows:

(41) $$FPR=\frac{FP}{FP+TN}$$

- Negative Predictive Value (NPV): This is a binary classification statistic that quantifies the percentage of actual negative instances among those that a model predicts to be negative. It has the following definition:

(42) $$NPV=\frac{TN}{TN+FN}$$

This pipeline outlines the systematic approach to handling data for machine learning tasks. It starts with inputting raw data, followed by preprocessing steps such as lowering text, removing stop words, and tokenizing and lemmatizing the content. Feature extraction techniques like TF-IDF and bag-of-words transform the processed data into numerical representations. A Stratified Shuffle Split ensures balanced train–test splits, crucial for maintaining class proportions in classification tasks. The models are then trained on the training set. Evaluation metrics like accuracy score, confusion matrix, and classification reports assess model performance, allowing for iterative improvements. Finally, successful models are deployed for real-world applications, completing the cycle of transforming raw data into actionable insights.

## 4. Results

**Figure 2:**
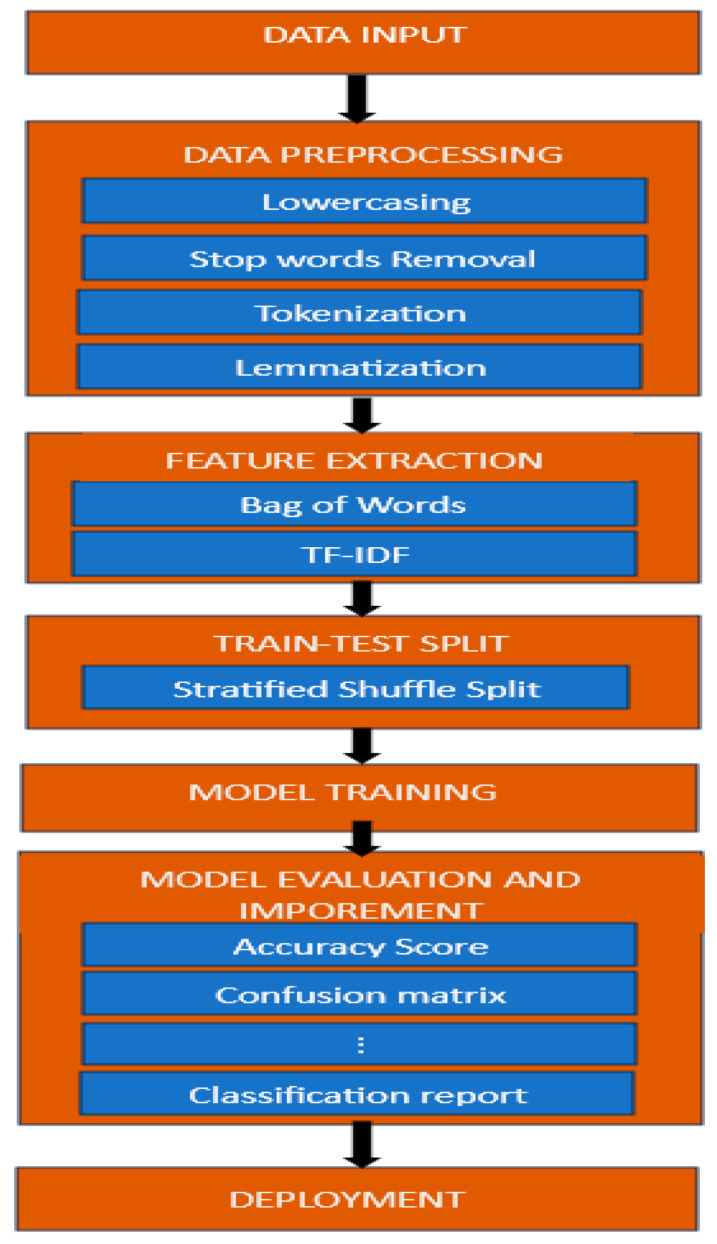
Architecture of fake news detection model.

The architectural model of the proposed system is depictured in Figure 2. The Sci-kit learn module and the open-source Python programming language were used to create the models used in the experiment. Jupyter Notebook was used as the implementation and testing environment of choice. More information covering this is provided in a later section. Throughout this project, a variety of machine learning algorithms which can be categorized into traditional and ensemble classifiers were employed, including “lbfgs” logistic regression, “liblinear” logistic regression, “newton-cg” logistic regression, “sag” logistic regression, Random Forest, perceptron, Ridge Classifier, CatBoost, Nearest Centroid, Stochastic Gradient Descent (SGD), SVC (kernel = “linear”, C = 0.025), SVC (gamma = 2, C = 1), LinearSVC, ZeroR, decision tree, Passive Aggressive, Extra Tree, Random Patches, voting, stacked generalization, multilayer perceptron (MLP), Bernoulli RBM, AdaBoost, gradient boosting, ordinal learning model (OLM), XGBoost, decision stump, Complement Naïve Bayes, and Multinomial Naïve Bayes, which were thoroughly trained using our four datasets, and each algorithm produced outputs. Every algorithm that considered our performance metrics produced an output result that comprised the following, as was stated in chapter three: F1 Score, MCC, KAPPA, accuracy, specificity, precision, recall, FDR, FPR, FNR, and NPV. We can determine which classifier performs best at recognizing fake news based on the accuracy levels.

### 4.1. Data Analysis of Dataset 1

Table 3 displays the outcome of all supervised machine learning algorithms used for Dataset 1, stacked generalization, which is an ensemble algorithm that had the maximum accuracy score of 0.9805 overall. The highest accuracy score achieved by a traditional learning algorithm was attained by the Ridge Classifier with a value of 0.9776. ZeroR performed the poorest overall, accuracy-wise. ComplementNB achieved the highest overall precision score at 0.9959. With a precision value of 0.9863, the voting classifier was the model with the highest ensemble value.

### 4.2. Data Analysis of Dataset 2

With an accuracy score of 0.9991, Bernoulli RBM had the best level of accuracy among all the algorithms, and according to the data in Table 4, decision stump, with an accuracy rating of 0.9962, was the model with the highest value. ZeroR had the greatest recall value of 1.0000, but its accuracy score was a pitiful 0.5229. The Bernoulli RBM, with a value of 0.9960, comes next. At 0.9432, Complement NB has the lowest recall value.

### 4.3. Data Analysis of Dataset 3

Out of all the algorithms utilized, the stacked generalization algorithm had the best accuracy and the fourth-highest precision scores. In terms of precision, Random Patches have the greatest value.

In terms of recall, the stacked generalization classifier algorithm appears to be the best choice, with a value of 0.9707. With ZeroR, the lowest accuracy of 0.5143 is attained. With a precision value of 0.0000, ZeroR produced the lowest result. Additionally, ZeroR was also thought to be the lowest, having the lowest recall score of 0.0000. The results on datasets 3 are provided in Table 5.

### 4.4. Data Analysis of Dataset 4

The stacked generalization algorithm had the highest accuracy score of all the algorithms used. The next four machine learning algorithms, “lbfgs”, “liblinear”, “newton-cg”, and “Sag” logistic regression, all had the same accuracy rating of 0.6088. According to data in Table 6, the voting classifier’s accuracy was the lowest. SVC (gamma = 2, C = 1) had the maximum precision score of 0.6053. Gradient boosting comes in second with a score of 0.5943, and Multinomial NB comes in third with a number of 0.5994. With a score of 0.0000, SVC (kernel = “linear”, C = 0.025), ZeroR, and decision stump obtained the lowest results.

The three models with the lowest recall scores were MLP, Extra Tree, and Random Forest, at 0.3797, 0.3619, and 0.3530, respectively. With a score of 0.68, the Bernoulli RBM performed the best, followed by the Nearest Centroid algorithm (0.6269) and the decision tree (0.5178).

## 5. Prediction of the Best-Performing Algorithms

**Figure 3:**
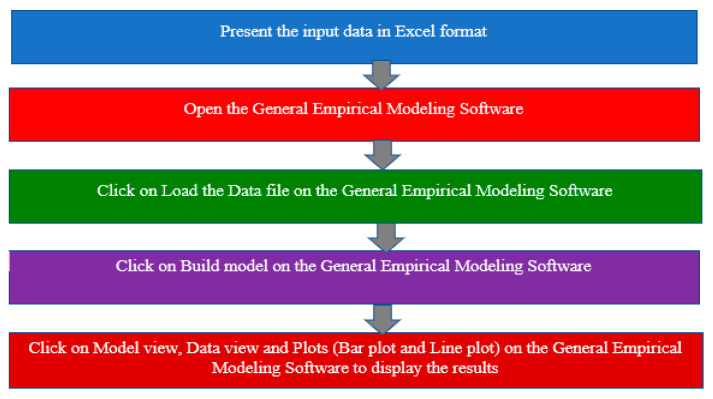
Stages involved in general empirical modeler (GEM 16.0).

**Figure 4:**
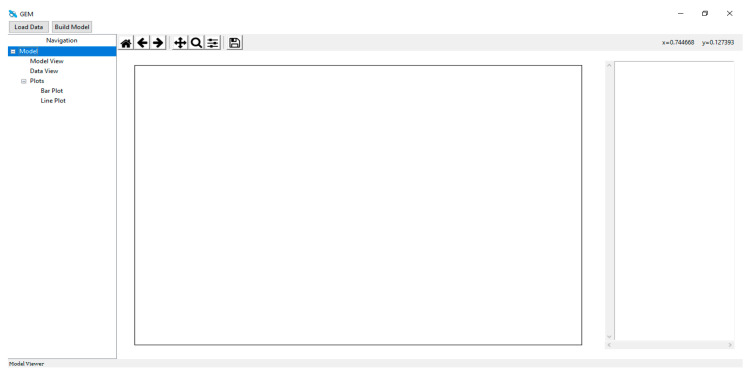
Graphical user interface of general empirical modeler (GEM 16.0).

Different models with high accuracy have been developed for the determination of the characteristics of the composites. The complexities and difficulties involved in the applications and computations of these models make them unsuitable to be used in practice. Therefore, in this work, a relatively simple approach using general empirical modeler (GEM 16.0) software was adopted. It is simple mathematical modeling software with great accuracy and ease of computation, mainly developed for the prediction of multiple quality characteristics. The procedures (the flowchart) and the graphical user interface of the software are given in Figure 3 and Figure 4.

In an attempt to obtain the best-performing algorithms across the given datasets, a predictive model was developed (see Equation (43)).

PREDICTIVE MODEL = 0.9890 × ACC^49.2552^ × PR^2.0131^ × RE^1.0522^ × SP^1.0174^ × FS^1.8945^ × MC^−0.1147^ × KA^1.4140^ × AU^−48.5261^ × FP^0.9440^ × FN^0.9172^ × FD^1.1328^ × NV^1.7497^ (43)

Here, AC is the accuracy, PR is the precision, RE is the recall, SP is the specificity, FS is the F1 Score, MC is the Matthews correlation coefficient (MCC), KA is the Cohen’s Kappa score, AU is the Area Under the Curve (AUC), FP is the false positive rate (FPR), FN is the false negative rate, FD is the False Discovery Rate, and NV is the Negative Predictive Value (NPV).

From Equation (43), the input for each of the defined parameters above was taken from Table 3, Table 4, Table 5 and Table 6. The corresponding (predicted) values were later obtained through the model. This technique has undoubtedly scaled down the choice of the best-performing algorithms from each of the datasets. 

**Figure 5:**
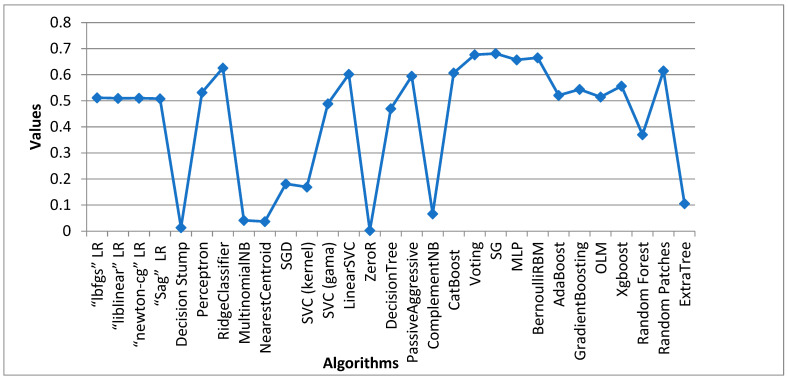
Predicted values for the algorithms with Dataset 1.

**Figure 6:**
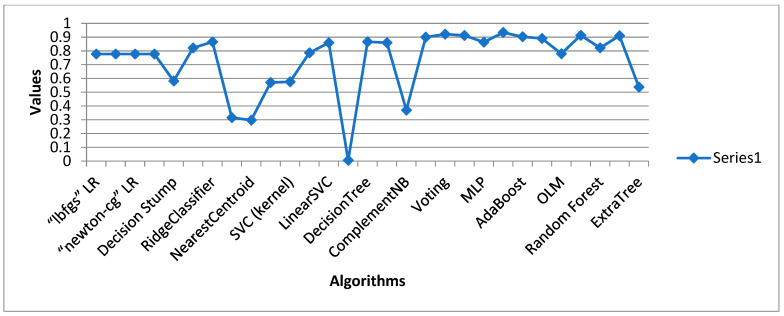
Predicted values for the algorithms with Dataset 2.

**Figure 7:**
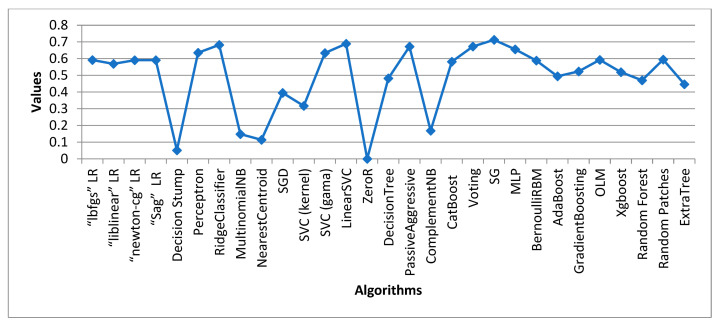
Predicted values for the algorithms with Dataset 3.

**Figure 8:**
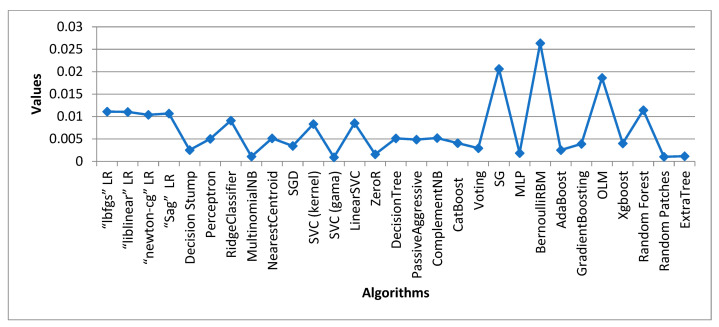
Predicted values for the algorithms with Dataset 4.

The results obtained for each of the datasets are presented in Table 7, while the graphical representations are shown in Figure 5, Figure 6, Figure 7 and Figure 8. With the predictive model developed, it is very easy to identify the best-performing algorithm for each of the datasets. The analysis is very easy and much more descriptive.

The accuracy results of different classifiers in classification tasks are shown in Table 8. Out of all the examples in the dataset, the accuracy column shows the percentage of correctly identified instances. From the foregoing, the new approach as shown on Table 8 has the highest level of accuracy. In most cases, some of the compared similar works used only accuracy as a metric for benchmarking the efficiency of the works. Suffice it to say that this approach as adopted by these researchers confirms that the results obtained in our case are more reliable and efficient than those of other similar researchers. The results obtained in [41,42,43,44,45,46,47] as well as the results by Khan et al., 2021 [48] confirm the underperformance of their techniques as presented in this study.

## 6. Discussion and Conclusions

The issue of “fake news” is one of the primary issues with the development of technology (the internet), social media, and other online activities. As was covered in chapter two, it can be used by evildoers for evil intentions. In order to identify fake news on social media, this study suggests a method that combines text mining with supervised AI algorithms [49]. Text mining analysis and supervised artificial intelligence algorithms have been the subject of independent research. This combination model was tested using four different real-world datasets, and the outcomes were examined using the following metrics: MCC, KAPPA, F1 score, accuracy, specificity, recall, precision, FPR, FNR, FDR, and NVP [50]. 

A total of four different datasets were used to train the machine learning classifiers for determining whether a news item is authentic or fake. Extensive experiments were used to train all supervised machine learning techniques, such as “lbfgs” logistic regression, “liblinear” logistic regression, “newton-cg” logistic regression, “sag” logistic regression, Random Forest, perceptron, Ridge Classifier, CatBoost, Nearest Centroid, Stochastic Gradient Descent (SGD), SVC (kernel = “linear”, C = 0.025), SVC (gamma = 2, C = 1), LinearSVC, ZeroR, decision tree, Passive Aggressive, Extra Tree, Random Patches, voting, stacked generalization, multilayer perceptron (MLP), Bernoulli RBM, AdaBoost, gradient boosting, ordinal learning model (OLM), XGBoost, decision stump, Complement Naïve Bayes, and Multinomial Naïve Bayes, which were trained through extensive experiments. Table 3, Table 4, Table 5 and Table 6 provide detailed results about their performance on four different datasets. 

A model based on optimized regression was developed to identify the best-performing algorithm across each of the four datasets. The results are provided in Table 7. The results obtained show SG in Dataset 1, with 0.681190 as the highest value. In Dataset 2, Bernoulli RBM has the highest value of 0.933789, while LinearSVC has the highest value of 0.689180 in Dataset 3. Finally, BernoulliRBM has the highest value of 0.02634 in Dataset 4.

In summary, the aim and objectives the authors set out to achieve in this research have been successfully achieved. This is visible from the results that have been reported. Future research could enhance the current work by incorporating the detection of bogus news using both image and video sources. Collection and processing of data through sensors have a crucial role as they can be utilized to capture real-time data from various social media platforms, facilitating continuous monitoring and supporting the implementation of AI algorithms and big data analytics in smart city initiatives [51]. 

Advanced machine learning and AI techniques such as explainable AI (XAI) and deep learning models, multimodal analysis such as cross-platform analysis, and natural language processing (NLP) improvements that have multilingual capabilities as well as a technique that will give room for real-time detection of fake news across the globe are the issues that are of future consideration in this line of research [52].

The major shortcoming of this technique is its failure, at the moment, to be classified as misleading or wrong. This is the most effective method for ensuring the accuracy of the stories under consideration. Achieving this will undoubtedly add to the quality and reliability of the results being generated, apart from flagging them as good or bad based on what is seen or observable.

Sufficient computational resources are available to facilitate algorithmic processing, but large amounts of data are required for continual training and testing of algorithms. 

While skilled producers of fake news may devise evasion strategies, algorithms are bound to inherit biases found in training data. Furthermore, algorithms could have trouble comprehending complex settings. For algorithms to be effective, real-time information processing is required. It is crucial to encounter a scenario in which the algorithms might not function properly in different linguistic and cultural contexts. Algorithms have a natural tendency to classify news as true or phony in error.

Finally, this work adopted a relatively simple approach for the prediction of the best-performing algorithms using general empirical modeler (GEM 16.0) software developed by [53].

## Figures and Tables

**Table 1:** Dataset source.

Dataset	URL	Source	Remark
Dataset 1	https://www.kaggle.com/competitions/fake-news/data (accessed on 21 March 2024)	Kaggle	Kaggle.com
Dataset 2	https://onlineacademiccommunity.uvic.ca/isot/2022/11/27/fake-news-detection-datasets (accessed on 23 March 2024)	Onlineacademic community.uvic.ca	onlineacademiccommunity.uvic.ca
Dataset 3	https://paperswithcode.com/dataset/liar (accessed on 25 March 2024)	paperswithcode	paperswithcode.com
Dataset 4	https://zenodo.org/record/4561253 (accessed on 22 March 2024)	Zenodo	Zenodo.org

**Table 2:** The confusion matrix.

	PREDICTED NEGATIVE (0)	PREDICTED POSITIVE (1)
**ACTUAL NEGATIVE (0)**	TRUE NEGATIVE (TN)	FALSE POSITIVE (FP)
**ACTUAL POSITIVE (1)**	FALSE NEGATIVE (FN)	TRUE POSITIVE (TP)

**Table 3:** Results of Dataset 1.

Model	Acc	Prec	Rec	Spec	F1 Score	MCC	KAPPA	AUC	FPR	FNR	FDR	NVP
“lbfgs” LR	0.9627	0.9599	0.9699	0.9595	0.9629	0.9254	0.9254	0.9627	0.9225	0.9226	0.9227	0.9656
“liblinear” LR	0.9627	0.9599	0.9659	0.9595	0.9629	0.9254	0.9254	0.9627	0.9225	0.9221	0.9235	0.9656
“newton-cg” LR	0.9627	0.9599	0.9659	0.9595	0.9629	0.9254	0.9254	0.9627	0.9225	0.9215	0.9245	0.9656
“Sag” LR	0.9627	0.9599	0.9659	0.9595	0.9629	0.9254	0.9254	0.9627	0.9225	0.9205	0.9225	0.9656
Decision Stump	0.7745	0.7242	0.8876	0.661	0.7976	0.5634	0.5488	0.7743	0.6111	0.6511	0.6147	0.8543
Perceptron	0.9668	0.9642	0.9697	0.9638	0.9669	0.9336	0.9336	0.9668	0.9211	0.9221	0.9211	0.9694
Ridge Classifier	0.9776	0.9756	0.9798	0.9754	0.9777	0.9552	0.9552	0.9776	0.9334	0.9314	0.9354	0.9796
Multinomial NB	0.8543	0.9959	0.7119	0.9971	0.8303	0.7395	0.7287	0.8545	0.6111	0.6101	0.6144	0.7753
Nearest Centroid	0.8463	0.7874	0.9495	0.7428	0.8609	0.7079	0.6926	0.8462	0.6133	0.6123	0.6153	0.9362
SGD	0.9752	0.9741	0.9764	0.974	0.9753	0.9504	0.9504	0.9752	0.6233	0.6223	0.6236	0.9763
SVC (kernel = “linear”, C = 0.025)	0.9016	0.8552	0.9673	0.8358	0.9078	0.8103	0.8033	0.9015	0.8103	0.8111	0.8123	0.9623
SVC (gamma = 2, C = 1)	0.961	0.9549	0.9678	0.9542	0.9613	0.9221	0.9221	0.961	0.9121	0.9123	0.9221	0.9673
LinearSVC	0.9771	0.9769	0.9774	0.9768	0.9772	0.9543	0.9543	0.9771	0.9221	0.9231	0.9231	0.9773
ZeroR	0.5407	0.5507	0.5606	0.5604	0.6673	0.6073	0.6513	0.5323	0.6011	0.6211	0.6017	0.545
Decision Tree	0.9596	0.9614	0.9577	0.9614	0.9595	0.9192	0.9192	0.9596	0.9092	0.9082	0.9092	0.9577
Passive Aggressive	0.9762	0.9778	0.9745	0.9778	0.9761	0.9524	0.9524	0.9762	0.9221	0.9231	0.9221	0.9745
Complement NB	0.8536	0.9959	0.7105	0.9971	0.8293	0.7383	0.7073	0.8538	0.7071	0.7291	0.7471	0.7744
CatBoost	0.9737	0.9717	0.9759	0.9715	0.9738	0.9476	0.9475	0.9737	0.9375	0.9376	0.9375	0.9758
Voting	0.9802	0.9863	0.974	0.9865	0.9801	0.9606	0.9605	0.9802	0.9475	0.9495	0.9475	0.9743
SG	0.9805	0.9826	0.9783	0.9826	0.9805	0.961	0.961	0.9805	0.9465	0.9565	0.9465	0.9784
MLP	0.9764	0.981	0.9716	0.9812	0.9763	0.9529	0.9528	0.9764	0.9523	0.9534	0.9523	0.9718
Bernoulli RBM	0.9774	0.9769	0.9779	0.9768	0.9774	0.9548	0.9548	0.9774	0.9533	0.9533	0.9533	0.9778
AdaBoost	0.9634	0.9595	0.9678	0.959	0.9636	0.9269	0.9269	0.9634	0.9269	0.927	0.9269	0.9674
Gradient Boosting	0.9673	0.9655	0.9692	0.9653	0.9674	0.9346	0.9346	0.9673	0.9265	0.9266	0.9265	0.969
OLM	0.9627	0.9599	0.9659	0.9595	0.9629	0.9254	0.9254	0.9627	0.9254	0.9265	0.9254	0.9656
Xgboost	0.967	0.9637	0.9707	0.9634	0.9672	0.9341	0.9341	0.967	0.9341	0.9356	0.9341	0.9704
Random Forest	0.9331	0.9678	0.8963	0.9701	0.9307	0.8687	0.8663	0.9332	0.9304	0.9314	0.9304	0.9031
Random Patches	0.9728	0.9809	0.9644	0.9812	0.9726	0.9458	0.9456	0.9728	0.9441	0.9451	0.9441	0.9649
Extra Tree	0.9276	0.9816	0.9836	0.68	0.9234	0.8607	0.8553	0.9277	0.678	0.679	0.678	0.8844
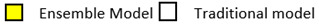												

LR—logistic regression. SGD—Stochastic Gradient Descent. SVC—support vector machine. SG—stacked generalization. MLP—multilayer perceptron. OLM—ordinal learning model.

**Table 4:** Results of Dataset 2.

Model	Acc	Prec	Rec	Spec	F1 Score	MCC	KAPPA	AUC	FPR	FNR	FDR	NVP
“lbfgs” LR	0.9853	0.9869	0.985	0.9857	0.9859	0.9706	0.9706	0.9853	0.9225	0.9226	0.9227	0.9836
“liblinear” LR	0.9853	0.9869	0.985	0.9857	0.9859	0.9706	0.9706	0.9853	0.9225	0.9221	0.9235	0.9836
“newton-cg” LR	0.9853	0.9869	0.985	0.9857	0.9859	0.9706	0.9706	0.9853	0.9225	0.9215	0.9245	0.9836
“Sag” LR	0.9853	0.9869	0.985	0.9857	0.9859	0.9706	0.9706	0.9853	0.9225	0.9205	0.9225	0.9836
Decision Stump	0.9962	0.9992	0.9936	0.9991	0.9964	0.9925	0.9925	0.9964	0.6111	0.6511	0.6147	0.9931
Perceptron	0.9905	0.9893	0.9925	0.9883	0.9909	0.9809	0.9809	0.9904	0.9211	0.9221	0.9211	0.9917
Ridge Classifier	0.994	0.9952	0.9932	0.9948	0.9942	0.988	0.988	0.994	0.9334	0.9314	0.9354	0.9926
Multinomial NB	0.9428	0.9448	0.9459	0.9394	0.9454	0.8855	0.8855	0.9427	0.6111	0.6101	0.6144	0.9406
Nearest Centroid	0.9375	0.9369	0.944	0.9303	0.9404	0.8747	0.8747	0.9371	0.6133	0.6123	0.6153	0.938
SGD	0.9927	0.994	0.9921	0.9935	0.993	0.9855	0.9855	0.9928	0.6233	0.6223	0.6236	0.9913
SVC (kernel = ”linear”, C = 0.025)	0.9703	0.9761	0.9668	0.974	0.9714	0.9405	0.9405	0.9704	0.8103	0.8111	0.8123	0.964
SVC (gamma = 2, C = 1)	0.9874	0.9877	0.9881	0.9865	0.9879	0.9747	0.9747	0.9873	0.9121	0.9123	0.9221	0.987
LinearSVC	0.9944	0.9944	0.9948	0.9939	0.9946	0.9888	0.9888	0.9944	0.9221	0.9231	0.9231	0.9943
ZeroR	0.5407	0.5507	0.5606	0.5604	0.6673	0.6073	0.6513	0.5323	0.6011	0.6211	0.6017	0.545
Decision Tree	0.9964	0.9968	0.9964	0.9965	0.9966	0.9929	0.9929	0.9964	0.9092	0.9082	0.9092	0.9961
Passive Aggressive	0.9944	0.9952	0.994	0.9948	0.9946	0.9888	0.9888	0.9944	0.9221	0.9231	0.9221	0.9935
Complement NB	0.9439	0.9492	0.9432	0.9446	0.9462	0.8876	0.8876	0.9439	0.7071	0.7291	0.7471	0.9381
CatBoost	0.9971	0.9996	0.9948	0.9995	0.9972	0.9942	0.9942	0.9972	0.9375	0.9376	0.9375	0.9944
Voting	0.9983	0.9984	0.9984	0.9982	0.9984	0.9966	0.9966	0.9983	0.9475	0.9495	0.9475	0.9982
SG	0.9975	0.9972	0.998	0.9969	0.9976	0.995	0.995	0.9975	0.9465	0.9565	0.9465	0.9978
MLP	0.9919	0.9952	0.9893	0.9948	0.9922	0.9839	0.9838	0.992	0.9523	0.9534	0.9523	0.9884
Bernoulli RBM	0.9991	0.9996	0.9988	0.9995	0.9976	0.9976	0.9983	0.9991	0.9533	0.9533	0.9533	0.9987
AdaBoost	0.9979	0.9992	0.9968	0.9991	0.9983	0.9983	0.9983	0.9979	0.9269	0.927	0.9269	0.9965
Gradient Boosting	0.9971	0.9992	0.9952	0.9991	0.9979	0.9942	0.9942	0.9972	0.9265	0.9266	0.9265	0.9948
OLM	0.9853	0.9869	0.985	0.9857	0.9859	0.9706	0.9706	0.9853	0.9254	0.9265	0.9254	0.9836
Xgboost	0.9987	0.9996	0.998	0.9995	0.9988	0.9975	0.9975	0.9987	0.9341	0.9356	0.9341	0.9978
Random Forest	0.9896	0.992	0.9881	0.9913	0.9901	0.9793	0.9793	0.9897	0.9304	0.9314	0.9304	0.987
Random Patches	0.9975	0.9992	0.996	0.9991	0.9976	0.995	0.995	0.9975	0.9441	0.9451	0.9441	0.9956
Extra Tree	0.9797	0.9915	0.9696	0.9909	0.9804	0.9597	0.9595	0.9802	0.678	0.679	0.678	0.9674
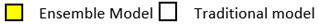												

LR—logistic regression. SGD—Stochastic Gradient Descent. SVC—support vector machine. SG—stacked generalization. MLP—multilayer perceptron. OLM—ordinal learning model.

**Table 5:** Results of Dataset 3.

Model	Acc	Prec	Rec	Spec	F1 Score	MCC	KAPPA	AUC	FPR	FNR	FDR	NVP
“lbfgs” LR	0.9615	0.9657	0.9547	0.968	0.9602	0.9231	0.9231	0.9614	0.9225	0.9226	0.9227	0.9577
“liblinear” LR	0.9615	0.9615	0.9615	0.9679	0.9229	0.9229	0.9229	0.9613	0.9225	0.9221	0.9235	0.9577
“newton-cg” LR	0.9615	0.9615	0.9547	0.9679	0.9613	0.923	0.9229	0.9613	0.9225	0.9215	0.9245	0.9577
“Sag” LR	0.9615	0.9615	0.9547	0.968	0.9602	0.9231	0.9231	0.9614	0.9225	0.9205	0.9225	0.9577
Decision Stump	0.8064	0.9771	0.6157	0.9863	0.7554	0.653	0.6084	0.801	0.6111	0.6511	0.6147	0.7311
Perceptron	0.9682	0.9682	0.9682	0.9696	0.9672	0.9364	0.9364	0.9682	0.9211	0.9221	0.9211	0.9686
Ridge Classifier	0.9728	0.9792	0.9646	0.9807	0.9718	0.9458	0.9457	0.9726	0.9334	0.9314	0.9354	0.967
Multinomial NB	0.8823	0.8622	0.9017	0.864	0.8816	0.7656	0.7648	0.8829	0.6111	0.6101	0.6144	0.903
Nearest Centroid	0.8662	0.8899	0.8267	0.9035	0.8267	0.7334	0.7316	0.8651	0.6133	0.6123	0.6153	0.8466
SGD	0.9606	0.968	0.9504	0.9703	0.9591	0.9214	0.9212	0.9604	0.6233	0.6223	0.6236	0.954
SVC (kernel = ”linear”, C = 0.025)	0.9209	0.9477	0.886	0.9539	0.9159	0.8432	0.8415	0.92	0.8103	0.8111	0.8123	0.8986
SVC (gamma = 2, C = 1)	0.9683	0.9762	0.9581	0.978	0.9671	0.9368	0.9367	0.9681	0.9121	0.9123	0.9221	0.9611
LinearSVC	0.9748	0.98	0.9678	0.9814	0.9739	0.9496	0.9496	0.9746	0.9221	0.9231	0.9231	0.97
ZeroR	0.5143	0	0	1	0	0	0	0.5	0.6011	0.6211	0.6017	0.5143
Decision Tree	0.9456	0.9513	0.9359	0.9548	0.9435	0.8912	0.8911	0.9453	0.9092	0.9082	0.9092	0.9404
Passive Aggressive	0.9726	0.9747	0.9688	0.9762	0.9717	0.9453	0.9453	0.9725	0.9221	0.9231	0.9221	0.9707
Complement NB	0.8807	0.8563	0.9065	0.8564	0.8807	0.7629	0.7617	0.8814	0.7071	0.7291	0.7471	0.9065
CatBoost	0.9586	0.9722	0.9417	0.9746	0.9567	0.9176	0.9172	0.9582	0.9375	0.9376	0.9375	0.9466
Voting	0.9703	0.9755	0.963	0.9772	0.9692	0.9406	0.9405	0.9701	0.9475	0.9495	0.9475	0.9655
SG	0.9755	0.9787	0.9707	0.98	0.9747	0.951	0.951	0.9753	0.9465	0.9565	0.9465	0.9725
MLP	0.9676	0.9714	0.9617	0.9733	0.9665	0.9353	0.9353	0.9675	0.9523	0.9534	0.9523	0.9642
Bernoulli RBM	0.9583	0.97	0.9433	0.9725	0.9565	0.9168	0.9165	0.9579	0.9533	0.9533	0.9533	0.9478
AdaBoost	0.946	0.953	0.935	0.9564	0.9439	0.8921	0.892	0.9457	0.9269	0.927	0.9269	0.9397
Gradient Boosting	0.9508	0.967	0.9304	0.97	0.9484	0.9021	0.9015	0.9502	0.9265	0.9266	0.9265	0.9366
OLM	0.9615	0.9656	0.9547	0.9679	0.9601	0.923	0.9229	0.9613	0.9254	0.9265	0.9254	0.9577
Xgboost	0.9494	0.9679	0.9266	0.971	0.9468	0.8995	0.8987	0.9488	0.9341	0.9356	0.9341	0.9334
Random Forest	0.9415	0.9499	0.9286	0.9537	0.9391	0.8831	0.8829	0.9412	0.9304	0.9314	0.9304	0.934
Random Patches	0.9599	0.9804	0.9361	0.9823	0.9577	0.9205	0.9197	0.9592	0.9441	0.9451	0.9441	0.9422
Extra Tree	0.9418	0.9299	0.9518	0.9323	0.9408	0.8839	0.8836	0.9421	0.878	0.8679	0.678	0.9535
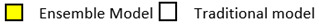												

LR—logistic regression. SGD—Stochastic Gradient Descent. SVC—support vector machine. SG—stacked generalization. MLP—multilayer perceptron. OLM—ordinal learning model.

**Table 6:** Results of Dataset 4.

Model	Acc	Prec	Rec	Spec	F1 Score	MCC	KAPPA	AUC	FPR	FNR	FDR	NVP
“lbfgs” LR	0.6088	0.5763	0.4075	0.766	0.4774	0.7231	0.9231	0.5868	0.9225	0.9226	0.9227	0.6234
“liblinear” LR	0.6088	0.5763	0.4075	0.766	0.4774	0.7229	0.9229	0.5868	0.9225	0.9221	0.9235	0.6234
“newton-cg” LR	0.6088	0.5757	0.4064	0.766	0.4765	0.723	0.9229	0.5862	0.9225	0.9215	0.9245	0.623
“Sag” LR	0.6083	0.5757	0.4064	0.766	0.4765	0.7231	0.9231	0.5862	0.9225	0.9205	0.9225	0.623
Decision Stump	0.5615	0.5615	0.5615	0.5615	0.5615	0.653	0.6084	0.5	0.6111	0.6511	0.6147	0.5615
Perceptron	0.5766	0.518	0.4944	0.6408	0.5059	0.7364	0.9364	0.5676	0.9211	0.9221	0.9211	0.6188
Ridge Classifier	0.5961	0.5456	0.4721	0.693	0.5062	0.7458	0.9457	0.5826	0.9334	0.9314	0.9354	0.627
Multinomial NB	0.5947	0.5994	0.2282	0.8808	0.4306	0.7656	0.7648	0.5545	0.6111	0.6101	0.6144	0.5937
Nearest Centroid	0.5834	0.5208	0.6269	0.5495	0.5689	0.7334	0.7316	0.5882	0.6133	0.6123	0.6153	0.6535
SGD	0.6049	0.561	0.4554	0.7217	0.5027	0.7214	0.9212	0.5885	0.6233	0.6223	0.6236	0.6292
SVC (kernel = ”linear”, C = 0.025)	0.5615	0.5615	0.5615	0.5615	0.5615	0.8432	0.8415	0.5	0.8103	0.8111	0.8123	0.5615
SVC (gamma = 2, C = 1)	0.6	0.6053	0.2527	0.8713	0.5566	0.8368	0.9367	0.562	0.9121	0.9123	0.9221	0.5989
LinearSVC	0.5859	0.5306	0.4821	0.6669	0.5052	0.8496	0.9496	0.5745	0.9221	0.9231	0.9231	0.6225
ZeroR	0.5615	0.5615	0.5615	0.5615	0.5615	0.5615	0.5615	0.5	0.6011	0.6211	0.6017	0.5615
Decision Tree	0.5556	0.4936	0.5178	0.5852	0.5054	0.8912	0.8911	0.5515	0.9092	0.9082	0.9092	0.6084
Passive Aggressive	0.5561	0.4939	0.4977	0.6017	0.4958	0.9453	0.9453	0.5497	0.9221	0.9231	0.9221	0.6054
Complement NB	0.6069	0.5686	0.4287	0.746	0.4888	0.7629	0.7617	0.5874	0.7071	0.7291	0.7471	0.6258
CatBoost	0.5937	0.5816	0.2616	0.853	0.3609	0.9176	0.9172	0.5573	0.9375	0.9376	0.9375	0.5967
Voting	0.5498	0.4866	0.4855	0.6	0.486	0.8406	0.9405	0.5427	0.9475	0.9495	0.9475	0.5989
SG	0.6123	0.5878	0.3875	0.7878	0.4671	0.851	0.951	0.5876	0.9465	0.9565	0.9465	0.6222
MLP	0.5571	0.495	0.3797	0.6008	0.498	0.7353	0.9353	0.5509	0.9523	0.9534	0.9523	0.6066
Bernoulli RBM	0.5947	0.5553	0.68	0.7626	0.451	0.9168	0.9165	0.5711	0.9533	0.9533	0.9533	0.6115
AdaBoost	0.5908	0.5877	0.2238	0.8773	0.3241	0.8921	0.892	0.5506	0.9269	0.927	0.9269	0.5914
Gradient Boosting	0.5942	0.5943	0.2349	0.8747	0.3367	0.9021	0.9015	0.5548	0.9265	0.9266	0.9265	0.5942
OLM	0.6083	0.5757	0.4064	0.766	0.4765	0.923	0.9229	0.5862	0.9254	0.9265	0.9254	0.623
Xgboost	0.5942	0.5943	0.2349	0.8747	0.3367	0.8995	0.8987	0.5548	0.9341	0.9356	0.9341	0.5942
Random Forest	0.6044	0.5805	0.353	0.8008	0.439	0.8831	0.8829	0.5769	0.9304	0.9314	0.9304	0.6131
Random Patches	0.5698	0.5149	0.3251	0.7606	0.3986	0.9205	0.9197	0.543	0.9441	0.9451	0.9441	0.5905
Extra Tree	0.5825	0.5354	0.3619	0.7547	0.4318	0.8839	0.8836	0.5583	0.678	0.679	0.678	0.6023
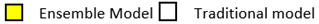												

LR—logistic regression. SGD—Stochastic Gradient Descent. SVC—support vector machine. SG—stacked generalization. MLP—multilayer perceptron. OLM —ordinal learning model.

**Table 7:** Comparative assessment of the actual values and predicted values.

Model	Predicted Values (Dataset 1)	Predicted Values (Dataset 2)	Predicted Values (Dataset 3)	Predicted Values (Dataset 4)
“lbfgs” LR	0.511607	0.777018	0.591782	0.011091
“liblinear” LR	0.509634	0.777017	0.568831	0.011035
“newton-cg” LR	0.509955	0.777017	0.590302	0.010385
“Sag” LR	0.508199	0.777019	0.590936	0.010678
Decision Stump	0.013878	0.581268	0.051723	0.002522
Perceptron	0.531805	0.821620	0.635029	0.005023
Ridge Classifier	0.626012	0.865666	0.682049	0.009085
Multinomial NB	0.041315	0.315335	0.148206	0.001084
Nearest Centroid	0.036675	0.296793	0.114558	0.005153
SGD	0.181611	0.570288	0.395300	0.003433
SVC (kernel)	0.169354	0.575824	0.317243	0.008315
SVC (gamma)	0.488523	0.785961	0.633574	0.0009135
LinearSVC	0.601572	0.858954	0.689180	0.008534
ZeroR	0.002557	0.005216	NAN	0.001566
Decision Tree	0.469843	0.866560	0.481940	0.005125
Passive Aggressive	0.594470	0.859099	0.672128	0.004876
Complement NB	0.066352	0.369456	0.168440	0.005208
CatBoost	0.606667	0.899864	0.581469	0.004068
Voting	0.676699	0.921623	0.672442	0.002935
SG	0.681190	0.912427	0.712431	0.020611
MLP	0.656821	0.863321	0.655427	0.001862
Bernoulli RBM	0.664927	0.933789	0.588572	0.026346
AdaBoost	0.520702	0.902569	0.494203	0.002534
Gradient Boosting	0.543842	0.890184	0.523236	0.003900
OLM	0.514580	0.779459	0.592771	0.018624
Xgboost	0.556446	0.912944	0.519105	0.003990
Random Forest	0.369743	0.822393	0.470116	0.011408
Random Patches	0.614726	0.910173	0.594396	0.001026
Extra Tree	0.105384	0.536867	0.446779	0.001154

**Table 8:** Fake and counterfeit news detection based on machine learning (ML) used for benchmarking the performance and computational overhead of the new approach.

Authors	Degree of Accuracy	Precision	Recall	ML Technique	Method Name
Wang 2017 [41]	25.5%	N/A	N/A	SVM	A new benchmark dataset for fake news detection
	27.0%	N/A	N/A	CNNs + LSTM	
	27.4%	N/A	N/A	CNNs + LSTM	
Long et al., 2017 [42]	25.5%	N/A	N/A	LSTM + Attention	Fake news detection through multi-perspective speaker profiles
	41.5%	N/A	N/A	LSTM + Attention	
Karimi et al., 2018 [43]	29.1%	N/A	N/A	CNN +LSTM	Multi-source multi-class fake news detection
	34.8%	N/A	N/A	CNN + LSTM	
Pham 2018 [44]	37.3%	N/A	N/A	Dual Attention	A study on deep learning for fake news detection
	44.2%	N/A	N/A	Memory Attention Network	
Kirilin and Strube 2018 [45]	41.5%	N/A	N/A	LSTM + Attention	Exploiting a speaker’s credibility to detect fake news
	45.7%	N/A	N/A	LSTM + Attention	
Liu et al., 2019 [46]	34.5%	N/A	N/A	2-Stage BERT_base + Attention	A two-stage model based on BERT for short fake news detection
	40.6%	N/A	N/A	2-Stage BERT_base + Attention	
Truong et al., 2020 [47]	61.0%	N/A	N/A	Bi-LSTM	Supervised classification methods for fake news identification
Khan et al., 2021 [48]	86.0%	86.0%	86.0%	CNN	A benchmark study of machine learning models for online fake news detection
New approach (Azeez et al., 2024)	98.05%	98.26%	97.83%	SG (Dataset 1) [21]	A predictive model for benchmarking the performance of algorithms for fake and counterfeit news detection in global networks
	99.9%	99.96%	99.88%	Bernoulli RBM (Dataset 2) [22]	
	97.55%	97.87%	97.07%	SG (Dataset 3) [24]	
	60.9%	57.63%	40.75%	“lbfgs” LR + “liblinear” LR + “newton-cg” LR (Dataset 4) [23]	

## Data Availability

The source code and datasets of the research are available at GitHub—rotex5/Fake_News_Detection.
